# Position and Strand Identity of G-Quadruplex Motifs Are Associated with Distinct Functional Signatures in Plants

**DOI:** 10.3390/ijms27219823

**Published:** 2026-11-02

**Authors:** Dmitry N. Kaluzhny, Daiana A. Krupskaya, Olesya D. Moskalenko, Elena N. Pushkova, Nataliya V. Melnikova, Alexey A. Dmitriev

**Affiliations:** Engelhardt Institute of Molecular Biology, Russian Academy of Sciences, 119991 Moscow, Russia; zhernova.d@ya.ru (D.A.K.); lesyamosk.bye-bye2003@yandex.ru (O.D.M.); pushkova18@gmail.com (E.N.P.); mnv-4529264@yandex.ru (N.V.M.)

**Keywords:** G-quadruplex, plant comparative genomics, homology, strand asymmetry, gene ontology enrichment

## Abstract

Putative G-quadruplex (G4) sequences have been linked to transcriptional regulation in individual plant species, but a systematic, comparative view across the plant kingdom has been lacking. In this study, we analyzed putative G4-forming sequences (PQS) near transcription start sites (TSSs) in 70 plant species spanning over 1.2 billion years of evolutionary divergence, from algae to angiosperms. Using the G4Hunter algorithm, putative G4 motifs were mapped and scored separately on the coding and template DNA strands. This strand-resolved analysis identified four position/strand categories, among which template-strand motifs downstream of the TSS were the most abundant and resistant to an increase in the G4Hunter threshold. The majority of PQS-positive genes (62.4%) carry a motif in only one category, although combinatorial patterns involving two or more categories also occur. Gene Ontology enrichment analysis of *Arabidopsis thaliana* homologs tested in each species revealed two main recurrent patterns of modest effect size: downstream template-strand motifs were associated with chloroplast and mRNA-binding terms, and upstream template-strand motifs were associated with transcription factor activity and regulation of transcription, whereas downstream coding-strand motifs showed a weaker signal. At the level of individual genes, position-specific G4s recurred among homologs more often than expected at random, mostly downstream, where this may partly reflect the conservation of the coding sequence. These associations, based on computational prediction, provide a starting point for prioritizing candidate G4 elements for experimental validation of their role in transcriptional control in plants.

## 1. Introduction

G-quadruplexes (G4s) are non-canonical, four-stranded secondary structures formed by stacked guanine tetrads (G-quartets) in guanine-rich DNA or RNA sequences, stabilized primarily by monovalent cations such as K^+^ [1]. Sequences with the potential to fold into G4 structures, known as putative G4-forming sequences (PQS), are widespread across eukaryotic genomes. In the human genome, early pattern-based searches already identified hundreds of thousands of such motifs, and the experimental G4-seq method later mapped over 700 thousand G4-forming regions genome-wide, including numerous non-canonical structures with long loops and bulges [2,3]. High-throughput chromatin-level mapping subsequently confirmed that G4s are not merely an in vitro phenomenon: BG4 antibody ChIP-seq detected roughly 10 thousand endogenous G4 structures in human chromatin, concentrated in regulatory, nucleosome-depleted regions, while G4access further linked G4 formation to open chromatin, active promoters, and imprinting control regions [4,5]. Consistent with this regulatory role, genome-wide chromatin profiling has shown that G4 sites act as hubs where multiple transcription factors converge, with a single G4 locus frequently co-occupied by dozens of distinct DNA-binding proteins [5]. Together, these data indicate that G4 motifs are non-randomly distributed across the genome, being enriched near transcription start sites (TSSs), in promoters, and in 5′ untranslated regions of actively transcribed genes.

Functionally, G4s can influence transcription by altering local chromatin accessibility, promoting nucleosome depletion, engaging sequence-specific regulatory proteins, and modulating promoter activity [6,7]. Detection of PQS has evolved accordingly: the original Quadparser algorithm relied on a rigid canonical motif (G_3+_N_1–7_G_3+_N_1–7_G_3+_N_1–7_G_3+_) [8], which is reproducible but misses a substantial fraction of non-canonical G4s with irregular G-tracts, bulges, or atypical loop lengths [2,6]. G4Hunter improved on this by replacing binary pattern matching with a continuous, sliding-window score reflecting G-richness and G/C skew, increasing sensitivity relative to canonical rules and suggesting that the number of potentially stable G4s in a genome may be 2 to 10-fold higher than previously estimated [9]. A subsequent web implementation made the algorithm broadly accessible for single-sequence queries [10]. However, the underlying scoring scheme is straightforward to reimplement directly, which is essential for analyses at the scale of this study, since genome-wide scanning across many species is not practical through a web interface designed for individual submissions. We therefore implemented the G4Hunter algorithm independently, following the original formulation [9], to enable batch prediction across full genome assemblies. More recently, machine-learning models such as Quadron, DeepG4, and G4Boost have incorporated experimental training data and additional sequence features, including loop composition and flanking context, to refine prediction further [11,12,13].

In plants, G4 maps remain available for only a handful of species, so comparative analyses across many genomes still rely predominantly on computational prediction. The first systematic survey, in *Arabidopsis thaliana*, identified roughly 1.2 thousand G3-type PQS (G_3+_N_1–7_ motifs, i.e., tracts of at least three consecutive guanines separated by loops of one to seven nucleotides), mostly intergenic, and around 43 thousand less stable G2-type PQS (G_2+_N_1–7_ motifs, with runs of only two consecutive guanines), a substantial fraction of which fell within genic regions. The associated Gene Ontology (GO) analysis found enrichment for enzymatic-activity categories and underrepresentation among non-coding RNA genes [14]. Takahashi et al. subsequently analyzed eight plant genomes, including *A. thaliana*, rice, poplar, and grape, and reported that the TSS region of the template strand was a consistent G4 hotspot in four of these species [15]. In rice specifically, this computational prediction was later corroborated at the chromatin level: genome-wide immunoprecipitation of DNA G4 structures confirmed their enrichment in genic regions and linked G4 occurrence to distinct epigenomic signatures and transcriptional regulation in living cells [16]. In *Vitis vinifera*, a recent genome-wide survey similarly found G4 enrichment in promoters and near TSSs, with a conservative strand preference. This strand bias was further linked to gene expression: genes upregulated under drought stress showed template-strand-biased G4 enrichment, while downregulated genes showed coding-strand dominance. G4-associated genes were implicated in sugar-acid metabolism and stress response [17]. In maize, G4 motifs were linked to genes involved in energy metabolism, hypoxia, and sugar/nutrient deprivation responses [18]. A 15-species comparison found that G3-type PQS were more common in promoters, introns, and intergenic regions, whereas G2-type PQS were enriched in gene bodies, CDS, exons, and UTRs, with conserved PQS-containing genes linked to development, cell growth, transmembrane transport, and gene expression regulation, and promoter PQS co-localizing with Telobox, ERF, MYB, RAV1B, and E2F motifs [19]. In wheat, near-genome-wide PQS were shown to be enriched at three specific locations, near the TSS on the antisense strand, in the first CDS on the antisense strand, and near the start codon on the sense strand, with associated genes enriched for developmental process, localization, and cellular component organization or biogenesis [20]. At the single-gene level, mechanistic evidence has begun to emerge as well: in *A. thaliana*, a template-strand G4 within *CYP82C4* was recently shown to be stabilized by the chromatin-remodeling factor TOP1α, directly repressing lateral root primordium initiation, a rare direct demonstration of G4-mediated gene regulation in a plant developmental context [21]. Beyond individual species, large-scale cataloguing efforts such as the Plant-GQ database have used canonical pattern-matching algorithms to predict PQS across nearly 200 plant genomes, but without resolving their position or strand relative to the TSS, or their association with gene function [22].

Yet such cases remain isolated examples, and existing comparative studies have three main limitations. First, most covered only a few species, so they could not show how G4 patterns evolve across the plant kingdom. Second, functional enrichment was usually assessed without separating G4 motifs by position and strand relative to the TSS, although G4s on the coding and template strands lie in different structural contexts during transcription and may differ in their functional associations. Third, GO enrichment depends on the PQS-detection algorithm, the stability threshold and the completeness of functional annotation, which makes studies hard to compare without a consistent pipeline applied uniformly across species.

Here, we address these gaps with a genome-wide, multi-species analysis of G4 motifs within a window around the TSS, spanning genomes from green algae to angiosperms. For each gene, a single, most-upstream TSS was used as the reference point, and G4 motifs were scored separately on the coding and template strands using G4Hunter across a threshold gradient, allowing position- and strand-resolved categorization of PQS at upstream/downstream on coding/template strand. G4 density patterns were visualized jointly with a time-calibrated phylogeny to track their evolutionary dynamics across more than 1.2 billion years of plant divergence. Functional associations of G4-containing genes were assessed via GO enrichment of their *Arabidopsis* homologs, and the recurrence of position-specific G4s among homologs was examined at the level of individual genes. This design allowed us to directly relate the recurrence of TSS-proximal G4 patterns to their position- and strand-specific functional signatures across the plant kingdom.

## 2. Results

### 2.1. Comparative Phylogenetic Analysis of the Spatial Distribution of Putative G-Quadruplexes Around Transcription Start Sites

To assess the cross-species recurrence and positional specificity of putative G-quadruplex sequences (PQS), we performed a genome-wide bioinformatics analysis spanning higher and lower plants, covering over 1.2 billion years of phylogenetic divergence across 70 species from the Phytozome database. G4 motif distribution was analyzed in a window around the transcription start site (TSS), separately for the coding (sense) and template (antisense) DNA strands, using the G4Hunter algorithm.

**Figure 1:**
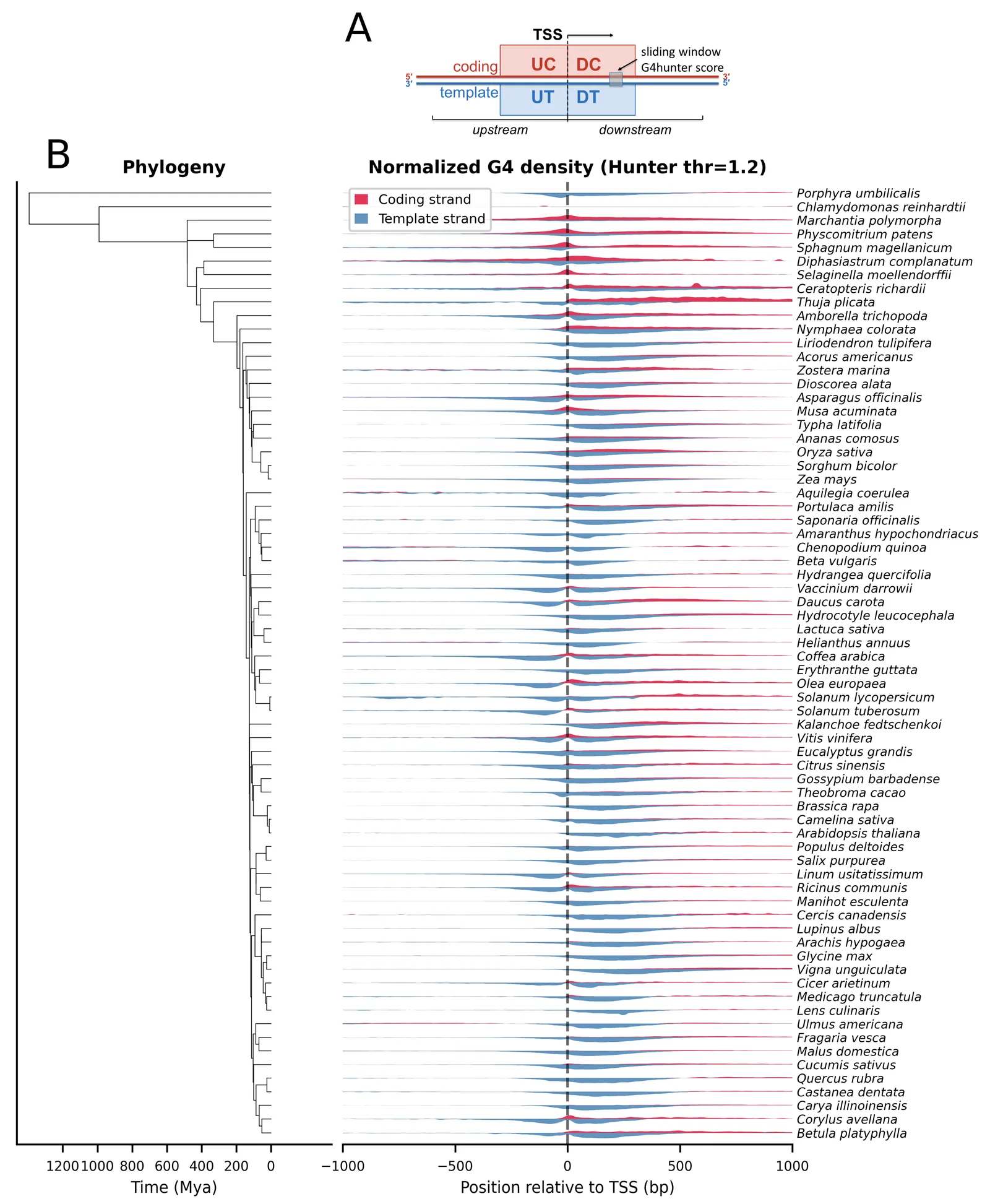
Evolutionary dynamics of G4 density profiles around transcription start sites (TSSs) across 70 plant species. (**A**) Schematic of the four position/strand categories relative to the TSS (UC—upstream coding, DC—downstream coding, UT—upstream template, DT—downstream template). (**B**) The time-calibrated phylogenetic tree (**left**), retrieved from the TimeTree database (http://timetree.org, accessed on 15 September 2026), is aligned with the normalized G4 density profiles within a ±1000 bp window relative to the TSS (**right**). G4 density at each position was normalized relative to a flanking periphery region (±1500–2000 bp from the TSS, outside the plotted range), the resulting scale is specific to each species. The coding strand is plotted above the baseline and the template strand below it; within each track, saturated red or blue indicates G4 enrichment relative to the periphery, corresponding to a higher probability of putative G4-forming sequences (PQS) occurrence than in the flanking region, while pale red or pale blue indicates depletion relative to the periphery, corresponding to a lower probability of PQS occurrence than in the flanking region. Normalized G4 density tracks were obtained using the G4Hunter algorithm with a threshold of 1.2 and a sliding window of 25 bp.

The analysis was performed across a gradient of G4Hunter thresholds (1.0, 1.4, 1.6, 1.8, and 2.0; Supplementary Figures S1–S5) to evaluate the robustness of the detected patterns. A threshold of 1.2 was selected as the baseline for detailed visualization (Figure 1) because it best discriminated experimentally tested G4-forming from non-forming genomic sequences in the original G4Hunter studies [9,10], with a false discovery rate (FDR) of about 10%.

Density profile analysis at a threshold of 1.2 revealed a clearly defined spatial compartmentalization of G4 motifs relative to the TSS position. In most angiosperm species examined, including both monocots, such as *Oryza sativa*, *Zea mays*, and *Sorghum bicolor*, and dicots, such as *Arabidopsis thaliana* and representatives of Fabaceae, Brassicaceae, and Solanaceae, a pronounced density peak of template-strand G4 motifs appears immediately downstream of the TSS, roughly within +100 to +500 bp. In the promoter region upstream of the TSS, the template-strand signal is substantially reduced, whereas in several taxa the coding strand instead forms a local peak immediately before the TSS, within the −200 to 0 bp window. Comparing these profiles along the phylogeny reveals several recurring patterns. Monocots and grasses of the family Poaceae show the highest density of template-strand G4 motifs within the coding region downstream of the TSS, around +50 to +400 bp, consistent with a possible role for G-quadruplexes in transcriptional pausing and RNA polymerase II elongation control in cultivated grasses. The fern *Ceratopteris richardii* and the gymnosperm *Thuja plicata* show an unusually high density of coding-strand G4 motifs across the entire analyzed window, with a pronounced bias toward the downstream region. In the green alga *Chlamydomonas reinhardtii*, the red alga *Porphyra umbilicalis*, and the bryophytes *Marchantia polymorpha* and *Physcomitrium patens*, the G4 signal at a threshold of 1.2 is more diffuse and concentrated directly at the TSS, which may reflect increasing complexity of regulatory G4 networks during the transition to vascular and seed plants.

These findings indicate that G4 localization in plant promoters and early transcribed regions is non-random. The strand asymmetry, with template-strand enrichment downstream of the TSS and coding-strand enrichment upstream in several taxa, is consistent with a possible role for quadruplexes in transcription initiation and elongation, which remains to be tested experimentally.

### 2.2. Genome-Wide Prevalence and Combinatorial Structure of PQS Categories

**Figure 2:**
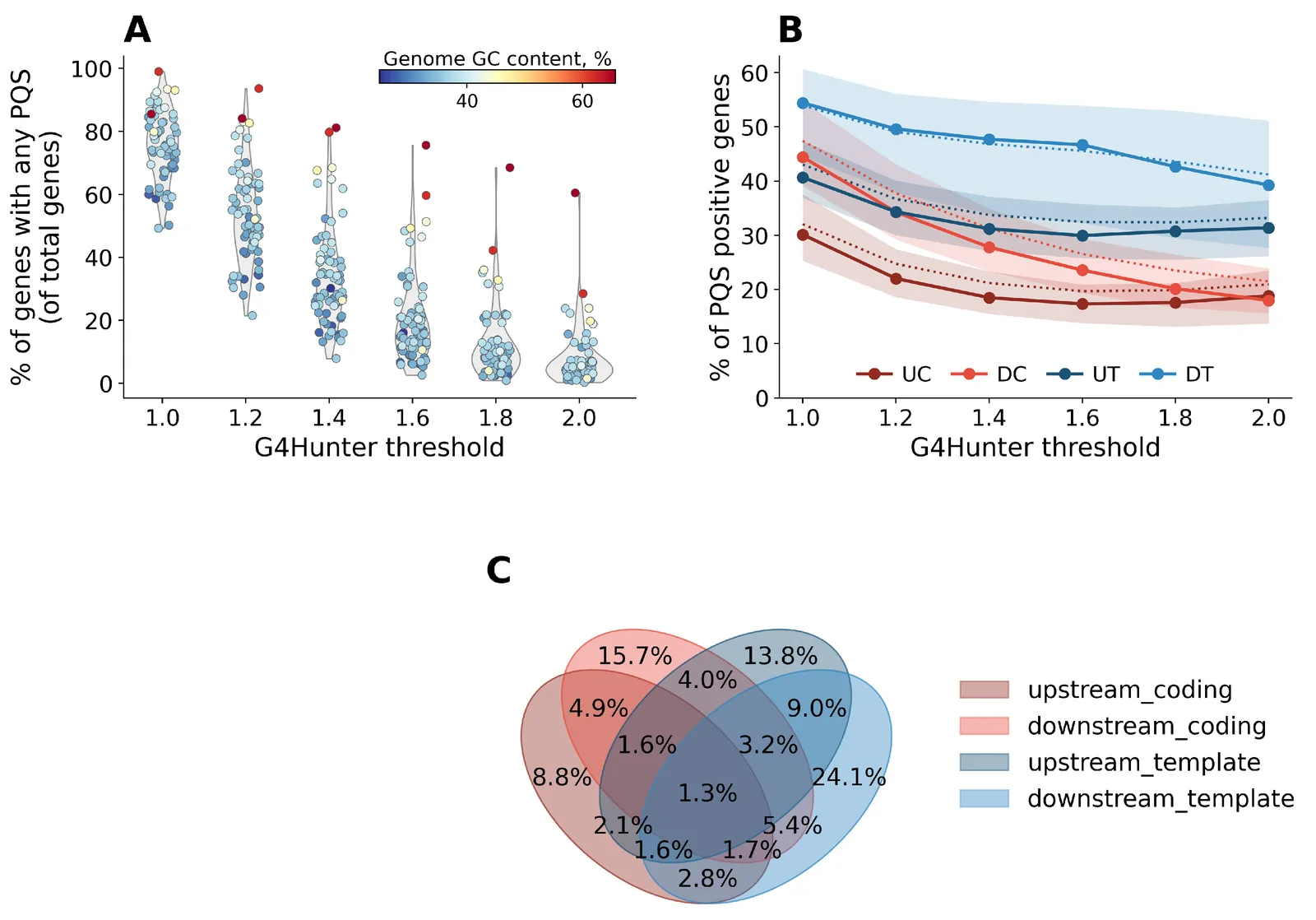
G4Hunter threshold sensitivity and combinatorial structure of putative G4-forming sequence (PQS)-positive genes. (**A**) Percentage of genes containing at least one predicted PQS within ±500 bp of the TSS, shown across a gradient of G4Hunter thresholds (1.0–2.0); each point represents one of the analyzed species, colored by genome GC content. (**B**) Relative proportions of the four position/strand categories (UC—upstream coding, DC—downstream coding, UT—upstream template, DT—downstream template) among all PQS-positive genes as a function of G4Hunter threshold. Solid lines show the median across species, dotted lines show the proportions calculated for all genes of all species pooled together, and semi-transparent bands show the interquartile range (25th–75th percentile across species). (**C**) Overlap between the four PQS categories among PQS-positive genes at a threshold of 1.2, shown as a four-way Venn diagram; percentages indicate the fraction of PQS-positive genes falling into each combination of categories.

To evaluate the fraction of the genome captured by PQS, and to characterize how this fraction depends on prediction stringency, we performed an aggregated analysis across all 70 plant species as a function of the G4Hunter threshold (Figure 2). The overall proportion of genes containing at least one PQS within ±500 bp of the TSS showed strong sensitivity to threshold choice, while preserving substantial inter-species variation (Figure 2A). At the least stringent threshold, the vast majority of genes genome-wide contained a PQS, with some species approaching near-complete coverage. At the baseline threshold of 1.2 used throughout this study, sequences with lower G4Hunter scores were filtered out, leaving roughly half of all genes PQS-positive, with considerable variation across species. This variation was associated with genomic GC content: genomes with a higher mean GC content had a higher probability that a gene contained a PQS (Spearman ρ = 0.59 at a threshold of 1.2; 0.54–0.62 across thresholds; Figure 2A, point color). At the most stringent threshold tested, the proportion of PQS-containing genes fell sharply, retaining only sequences with the highest G4Hunter scores.

All identified PQS were assigned to one of four categories based on their position and strand: upstream coding (UC), downstream coding (DC), upstream template (UT), and downstream template (DT). A gene can carry PQS of several categories. The proportion of PQS-positive genes carrying a motif of each category, pooled across all genes from all 70 species at each threshold, is shown in Figure 2B. Template-strand motifs downstream of the TSS (DT) predominated over all other categories across the entire threshold range, and this predominance became more pronounced as stringency increased. Coding-strand categories (DC and UC) showed the steepest decline with increasing threshold, i.e., template-strand motifs more often had high G4Hunter scores than coding-strand motifs. The UT category declined only modestly and plateaued at higher thresholds, in contrast to the steady erosion of the coding-strand categories.

To assess whether PQS tend toward category-exclusive localization or instead co-occur within a single gene, we analyzed the overlap among the four categories at the baseline threshold of 1.2 (Figure 2C). Genes carrying a PQS restricted to a single category comprised the majority, with the largest contributions from DT, DC, and UT, while UC contributed comparatively less. Pairwise overlaps between adjacent categories occurred at moderate frequency, while combinations involving three or all four categories at once were markedly rarer and may partly reflect motifs positioned near a category boundary rather than independent quadruplexes.

Together, these results indicate that the downstream template strand (DT) is not only the most abundant category but also the one most resistant to an increase of the G4Hunter threshold, i.e., it is enriched in motifs with high G4Hunter scores. The pronounced category exclusivity, with more than 60% of genes carrying a PQS in only one of the four sectors, suggests that PQS at different promoter and 5′-UTR positions are largely distinct features, consistent with possible position- and strand-specific roles.

### 2.3. Genes with and Without an Arabidopsis Homolog Differ Slightly in GC Content and G4Hunter Scores

To transfer functional annotation across 70 species, each protein-coding gene (longest transcript) was searched with BLASTp 2.12.0 against the *A. thaliana* proteome (TAIR10; E-value ≤ 1 × 10^−5^, best hit only). A hit was accepted as a homolog if it had ≥ 30% sequence identity and the alignment covered ≥ 50% of the query length; the filtering is summarized in Supplementary Figure S6. Excluding the *Arabidopsis* self-match, a median of 74% of genes per species had an accepted homolog (range 17–98%; Supplementary Figure S6A). The proportion was lowest in the two algae (*Porphyra umbilicalis*, 17%; *Chlamydomonas reinhardtii*, 22%) and exceeded 44% in all other species. Accepted hits had a median identity of 59% and covered a median of 98% of the query length. The identity of raw best hits was broadly distributed, with most hits well above the 30% cut-off (Supplementary Figure S6B; the peak at 100% is the *Arabidopsis* self-hit). The coverage criterion removed a median of 6.6% of otherwise acceptable hits per species, mostly short, partial alignments, typically matching a single domain.

**Figure 3:**
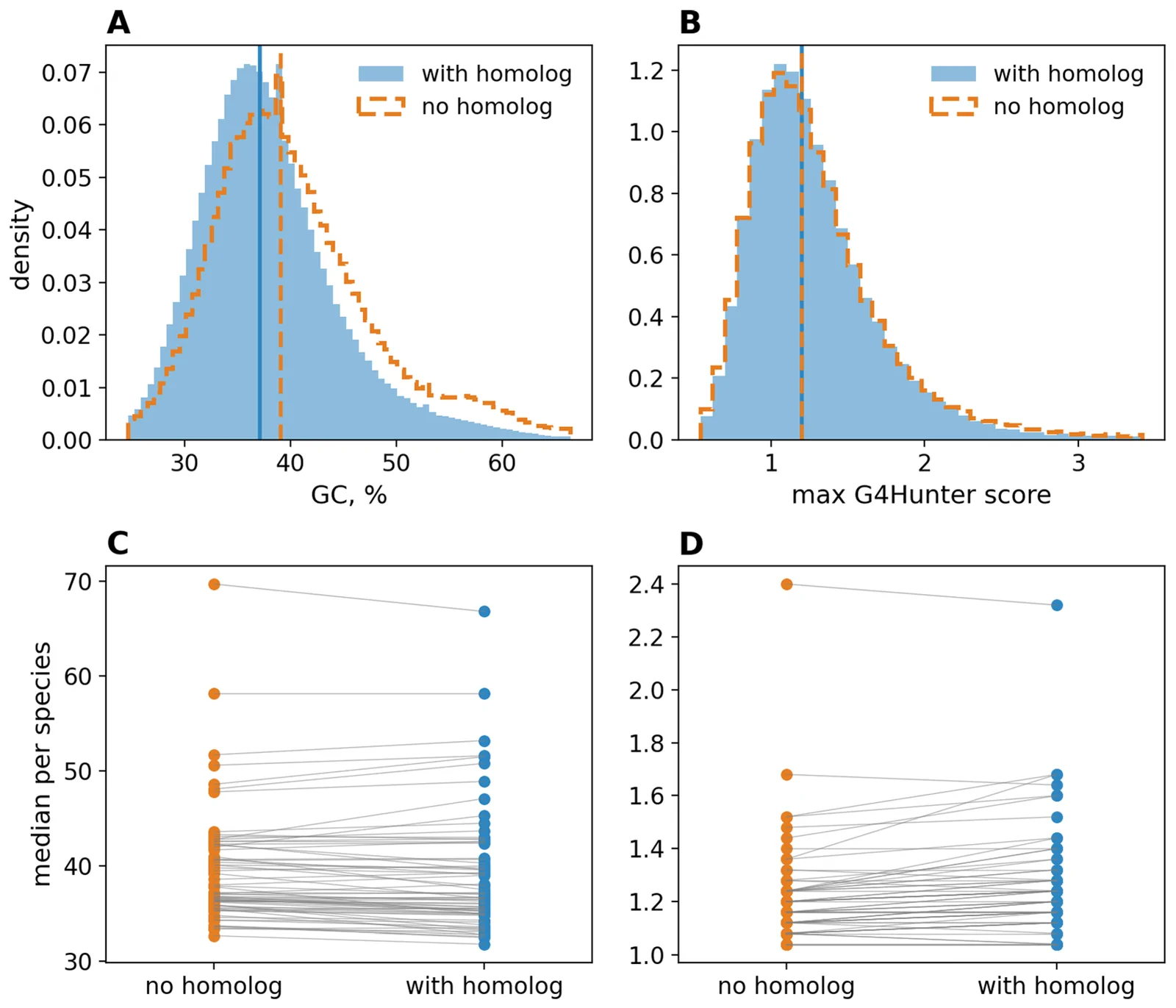
Sequence composition of genes with and without an *Arabidopsis* best-hit homolog. (**A**,**B**) Distributions of GC content (**A**) and maximum G4Hunter score (**B**) in the –500…+500 bp region around the TSS, pooled across 69 species (*Arabidopsis* was excluded). Blue filled bars: genes with a homolog; orange dashed outline: genes without a homolog. Vertical lines mark the medians. G4Hunter was computed with a 25 bp window. (**C**,**D**) Median value per species for the same two metrics; lines connect the two gene sets within one species.

The Gene Ontology (GO) analysis below is restricted to genes with an *Arabidopsis* homolog and uses the homolog-bearing genes of the same species as the statistical background. This raises a concern: genes without a homolog (lineage-specific, fast-evolving, or poorly annotated) may differ in sequence composition and G4Hunter score. GO enrichments could then reflect this difference rather than gene function. Therefore, we compared the two gene sets directly in the region around the TSS (–500 to +500 bp), using GC content and the maximum G4Hunter score per gene (Figure 3).

The two distributions largely overlap (Figure 3A,B). The medians of the GC content and the maximum G4Hunter score differed slightly between the two gene sets, and the difference had the same direction in most species (Figure 3C,D; paired Wilcoxon signed-rank test on species medians, *p* < 0.001). With the large number of genes per species, such differences are statistically detectable, but they are small compared to the variation between species. Because both the foreground and background of the GO analysis consist of genes with a homolog, this small shift does not bias the enrichment between categories. This comparison addresses only the bias associated with homolog assignment and does not exclude effects of nucleotide composition within the enrichment analysis itself; this is examined further in Section 2.4.

### 2.4. Gene Ontology Enrichment Differs Between Position/Strand Categories

To identify functional categories stably associated with G4 structures at specific positions relative to the TSS, we performed a systematic GO enrichment analysis across all studied plant species. To ensure cross-species functional comparability, *A. thaliana* homologs were first identified for each gene across all plant genomes, followed by GO enrichment testing against a background of the homolog-bearing genes of the same species.

To assess the phylogenetic stability of functional signatures associated with G4 motifs, we analyzed the recurrence of enriched GO terms across all studied plant species (Table 1). For each species, GO enrichment was evaluated independently within four position/strand categories: upstream coding (UC), downstream coding (DC), upstream template (UT), and downstream template (DT), using a species-specific background (FDR < 0.05; G4Hunter threshold = 1.2). Table 1 lists terms enriched in at least 20 species.

Cross-species comparison revealed distinct patterns tied to specific G4 topologies. Most prominently, terms related to chloroplast structure, such as chloroplast (GO:0009507), chloroplast stroma (GO:0009570), and chloroplast envelope (GO:0009941), recurred in 45–53 species, together with mRNA binding (GO:0003729; 43 species). This organellar signature was driven almost exclusively by downstream template-strand (DT) motifs. In contrast, key regulators of gene expression, including DNA-binding transcription factor activity (GO:0003700) and transcription cis-regulatory region binding (GO:0000976), formed a separate transcriptional control pattern. These terms were enriched in 57 species each, predominantly in the upstream template (UT) category, with a substantial contribution of downstream coding (DC) G4s and only rare enrichment in DT (10–12 species). Meanwhile, protein binding (GO:0005515) was enriched in 56 species with a more balanced representation across G4 categories. Effect sizes were modest (median fold enrichment 1.1–1.6), so these patterns indicate a recurrent association rather than a strong dependence. Together, they suggest that the association between G4 topology and specific functional programs is not restricted to a single lineage and recurs across the plant kingdom.

**Figure 4:**
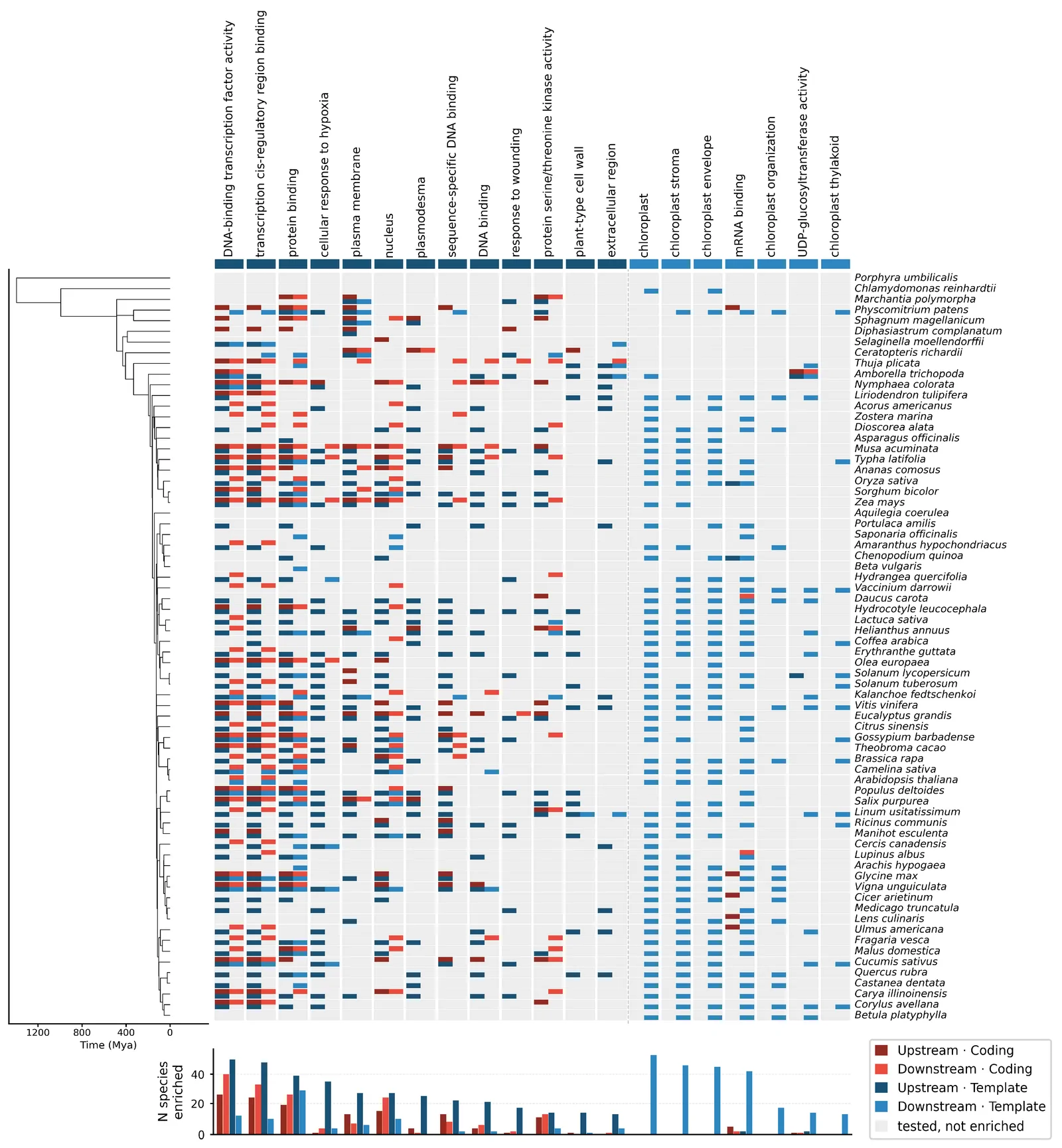
Phylogenetic distribution of recurrently enriched Gene Ontology (GO) terms among genes with a G4 motif near the transcription start site (TSS) (G4Hunter threshold = 1.2). Rows: 70 species on a time-calibrated tree; Columns: GO terms, grouped by the predominant category. Colored cells: term enriched (FDR < 0.05) in the given position/strand category; Gray: tested, not enriched. Bars below: number of species with enrichment in each category. Terms enriched in at least 15 species are shown; redundant terms (Jaccard index of the species sets > 0.6) are collapsed, so some terms in Table 1 (e.g., regulation of DNA-templated transcription) are not shown separately.

The phylogenetic distribution of the recurrent terms is shown in Figure 4. Terms related to transcription (DNA-binding transcription factor activity, transcription cis-regulatory region binding, sequence-specific DNA binding, and DNA binding) were enriched mainly among UT genes in species from across the tree; for the first two terms, DC and UC enrichment was also frequent. Cellular response to hypoxia, response to wounding, and the cellular component terms (plasma membrane, plasmodesma, plant-type cell wall, and extracellular region) were likewise UT-dominated, but in fewer species (15–36).

Chloroplast terms (chloroplast, chloroplast stroma, envelope, thylakoid, and chloroplast organization), mRNA binding, and UDP-glucosyltransferase activity formed a separate DT block, with almost no enrichment in the other categories. In contrast, protein serine/threonine kinase activity, nucleus, and protein binding were enriched in several categories without a clear preference; no term showed a DC-specific pattern comparable to UT or DT.

Thus, two patterns were the most consistent: UT enrichment of transcription-related terms and DT enrichment of chloroplast-related terms. These results are descriptive, since the species are not phylogenetically independent. The patterns were largely maintained at other G4Hunter thresholds (Supplementary Figure S7): transcription factor terms remained UT-enriched in 62–75% of the tested species at all thresholds, and chloroplast terms were stable up to 1.4, declining at higher thresholds as fewer species could be tested. Plasma membrane, plasmodesma, wounding, and hypoxia were less stable terms.

To test whether these associations reflect the presence of putative G4s rather than GC richness alone, we repeated the GO analysis after dividing the genes of each species into four quartiles by the GC content of the upstream and downstream windows (Supplementary Figure S8). Within each quartile, genes were split into those without a PQS (G4Hunter score < 1.2 on both strands) and those with a PQS on the coding strand or on the template strand (score ≥ 1.2). Each group, as well as all genes of the quartile, was tested against the background of all genes of the species with an *Arabidopsis* homolog. GC content alone partly reproduced the terms of Table 1, mainly in the GC-rich quartiles. However, the chloroplast-related terms (downstream, template strand) and the transcription-related terms (upstream, template strand; downstream, coding strand) were enriched in more species among PQS-positive genes than among all genes of the same GC quartile, and were largely absent from GC-matched genes without a PQS. A separate set of terms, such as plasma membrane, was associated with GC content irrespective of PQS status. Thus, GC content contributes to the observed associations but does not fully account for them.

### 2.5. Recurrence of Position-Specific G4s at Homologous Genes

To examine how the previously identified G4 signatures are represented at the level of individual genes, we next mapped G4 occurrence and topology across homologous gene groups in all 70 plant species. We considered the 7153 *Arabidopsis* genes with a homolog in at least 61 species and for each gene calculated the share of species whose homolog carries a G4 in each position/strand category. A gene was considered position-specific if a G4 was found in one category in at least a share T of the species and in no other category at that level. At T = 0.6, 984 genes were position-specific in DT, 197 in DC, 64 in UT, and 2 in UC. When G4-carrying genes were instead assigned at random within each species, 8, 0, 0, and 0 genes were expected on average (maximum in 1000 permutations: 19, 1, 1, and 0; Supplementary Figure S9). The excess over random was seen from T ≈ 0.5 for DT, DC, and UT. Genes with a recurrent downstream G4 were thus more than ten times more numerous than those with an upstream G4. Because homologs were identified from protein sequence similarity, the recurrence of downstream G4s may partly reflect the conservation of the amino acid sequence, and hence of the coding nucleotide composition, rather than selection on G4 structures; this explanation does not apply to upstream regions. The 66 genes with a recurrent upstream G4 are listed in Supplementary Table S2. They include transcription factors and genes of hormone metabolism and signaling, in line with the GO analysis. These tests are descriptive, since the species are not phylogenetically independent.

## 3. Discussion

Over the past decade, accumulating evidence has established that putative G-quadruplex-forming sequences (PQS) are biologically active regulatory features in plant genomes. Previous surveys across select model species, small plant cohorts, and individual gene targets demonstrated that G4 structures participate in transcriptional control, energy metabolism, developmental pathways, and stress responses [14,15,18,19,20,21,23]. However, because these prior insights were derived from isolated organisms, narrow taxonomic comparisons, or single-gene studies, the field lacked a unified evolutionary framework capable of explaining how G4 topology is organized across the plant kingdom.

Our work expands isolated, single-gene and single-species observations into a multi-species comparison. By analyzing 70 plant genomes spanning over 1.2 billion years of evolution, from unicellular green algae to angiosperms, with a threshold-gradient G4Hunter scheme and time-calibrated phylogenetics, we show that G4 position and strand identity are associated with distinct functional signatures, detected in the cross-species recurrence of GO terms (Table 1 and Figure 4). Downstream template-strand (DT) motifs were associated with chloroplast, organellar, and RNA-binding terms, which recurred across the deepest branches of the phylogeny examined here. Upstream template-strand (UT) motifs were instead associated with the transcriptional machinery itself, from DNA-binding transcription factor activity to the regulation of transcription, whereas stress-related terms such as response to hypoxia were less stable across thresholds. Downstream coding-strand (DC) motifs showed a weaker and less consistent signal, with no term specific to this category. The effect sizes of all enrichments were modest, so we regard these results as associations rather than as separate functional layers.

The billion-year conservation previously reported for the single *RPB1* locus [23] may therefore be representative of a broader class of recurrent G4 elements: the DT signature was associated with organellar and RNA-processing terms in species ranging from unicellular algae to angiosperms. At the gene level, the recurrence of a position-specific G4 among homologs was greater than expected at random (Supplementary Figure S9), but it was found mostly downstream, and very few genes carried a G4 in all of their homologs. Because the downstream window overlaps transcribed sequence, this recurrence may reflect the conservation of the coding sequence rather than selection on G4 structures. This explanation does not apply to upstream regions, where fewer genes were found; they included transcription factors and genes of hormone metabolism and signaling (Supplementary Table S2).

Several limitations should be noted. G4Hunter scores reflect the nucleotide composition of a sequence and not proven structure formation. G-rich template-strand motifs downstream of the TSS may partly arise from the amino acid composition of conserved proteins (for instance, serine-, proline-, or alanine-rich N-terminal regions of plastid-targeted proteins), rather than from selection on G4 structures. Homologs were assigned from protein similarity to *Arabidopsis thaliana*, which is incomplete for the most distantly related algae, and the species are not phylogenetically independent, so all statistical tests are descriptive. TSS positions were taken from the genome annotation (the most upstream annotated TSS of each gene) and may deviate from the actual start sites. Experimental TSS mapping [24] would allow the position categories to be defined more precisely. Composition-matched controls and experimental evidence will be needed to separate these explanations.

Establishing this multi-species map defines clear priorities for future research in plant structural genomics. Rather than relying solely on computational prediction, the field must now advance toward targeted in vivo structural profiling, such as G4-seq, CUT&Tag, and DMS footprinting, focused specifically on the high-enrichment functional gene families identified in this study. Resolving the precise positional and strand topology around the TSS also provides a structural blueprint for investigating how chromatin remodelers, such as TOP1α [21], and single-stranded nucleic acid-binding proteins interact with dynamic quadruplexes to tune transcriptional kinetics.

Finally, if confirmed experimentally, the position- and strand-specific associations described here might help to prioritize G4 elements for targeted modification in crop plants. Because G4 formation depends on the local sequence and on cellular conditions, such elements could in principle be used to tune the expression of stress-related or metabolic genes, but this possibility is speculative and requires experimental validation.

## 4. Materials and Methods

### 4.1. Plant Genome Data and Species Selection

Genome assemblies and gene annotations (GFF3) for 70 plant species were obtained from Phytozome v13 [25], spanning unicellular green and red algae, bryophytes, lycophytes, ferns, gymnosperms, and angiosperms. Species were selected to provide broad taxonomic coverage of the plant kingdom, subject to the availability of a genome assembly and structural annotation.

### 4.2. Prediction of Putative G-Quadruplex Sequences and Position/Strand Categorization

For each gene, the sequence spanning 2000 bp upstream and downstream of the most upstream annotated transcription start site (TSS) was extracted, using a single representative transcript per gene. Putative G4-forming sequences (PQS) were predicted with a custom implementation of the G4Hunter algorithm [9]: each guanine within a G-run was assigned a score equal to its run length (capped at 4), each cytosine within a C-run was assigned the corresponding negative value, and these per-base scores were averaged over a 25 bp sliding window. Windows with a G4Hunter score above a given threshold were classified as coding-strand PQS, and windows scoring below the corresponding negative threshold as template-strand PQS. Here, the coding (sense) strand is the non-template strand, whose sequence is identical to the mRNA, and the template (antisense) strand is the strand read by RNA polymerase (Figure 1A). Therefore, a positive G4Hunter score denotes a G-rich coding strand and a negative score a G-rich template strand. The sequence of each gene was oriented according to the direction of transcription before the analysis: for genes on the negative genomic strand, the extracted region was reverse-complemented, so that the TSS was at position 0, and all sequences were analyzed in the direction of transcription. G4Hunter was then calculated on the oriented sequence in the same way for all genes. Prediction was repeated across a threshold gradient (1.0–2.0 in steps of 0.2), with 1.2 used as the baseline threshold throughout the study. Each gene was further classified into one or more of four non-exclusive position/strand categories, upstream_coding (UC), downstream_coding (DC), upstream_template (UT), and downstream_template (DT), based on whether a PQS was detected within 500 bp upstream or downstream of the TSS on the coding or template strand. The downstream window therefore overlaps the transcribed, and in part the protein-coding, sequence.

### 4.3. Phylogenetic Reconstruction and Time Calibration

A time-calibrated phylogeny for all 70 species was retrieved from the TimeTree database [26] (https://timetree.org/, accessed on 15 September 2026) and used to order species and assign divergence times (in millions of years) throughout the study. Species names were matched between the phylogeny and the genomic dataset using a manually curated synonym table to resolve taxonomic naming discrepancies.

### 4.4. Homology Mapping to Arabidopsis thaliana

Predicted proteomes (longest transcript per gene) of all 70 species were searched against the *A. thaliana* (TAIR10) [27] proteome using BLASTp 2.12.0 [28] with -max_target_seqs 1 and one local alignment per query, and the single best hit per query gene (highest bitscore) was retained. Hits were filtered using an E-value threshold of 10^−5^, a minimum sequence identity of 30%, and a minimum query coverage of 50%. The *Arabidopsis* gene of the retained hit was referred to as the homolog of the query gene. This assignment does not distinguish orthologs from paralogs, because it was based on best-hit protein similarity. Homology assignment quality was assessed per species from the number of retained hits and their average percent identity (Supplementary Figure S6). GC content and the maximum G4Hunter score per gene in the −500…+500 bp region around the TSS were compared between genes with and without an accepted homolog.

### 4.5. Gene Ontology Enrichment Analysis

For each species and each of the four PQS categories, Gene Ontology (GO) enrichment [29] of the *Arabidopsis* homologs of PQS-carrying genes was tested using a one-sided Fisher’s exact test with Benjamini–Hochberg [30] false discovery rate (FDR) correction. The background was species-specific: it comprised all *Arabidopsis* genes with a TAIR GO annotation that were identified as homologs of genes of that species, so that enrichment was not influenced by differences in the proportion of genes with detectable homologs between species. Terms represented by fewer than three foreground genes were excluded, and categories with fewer than 300 foreground genes with GO annotation in a given species were not tested. Terms with FDR < 0.05 were considered significantly enriched. The analysis was repeated for each G4Hunter threshold from 1.0 to 2.0.

### 4.6. Cross-Species Recurrence of GO Terms

A GO term was considered recurrent in a PQS category if it was significantly enriched in at least 20 of the 70 species at the baseline G4Hunter threshold of 1.2 (Table 1). The category pattern of a term was defined as DT or UT if that category accounted for at least 60% of the species in which the term was significant, and as mixed otherwise. For the species × term heatmap (Figure 4), terms significant in at least 15 species were shown, and redundant terms (Jaccard index of the species sets > 0.6) were collapsed into one; the heatmap was drawn alongside the time-calibrated phylogeny. The stability of each term across thresholds was expressed as the percentage of species, among those in which the category could be tested (≥300 foreground genes with GO annotation), in which the term was significant (Supplementary Figure S7).

### 4.7. Gene-Level Permutation Test

To test whether G4s recur at homologous genes more often than expected by chance, we considered the *Arabidopsis* genes with a homolog in at least 61 of the 70 species (7153 genes). For each gene and each category, we calculated the share of species with a homolog in which that homolog carried a PQS in the category (G4Hunter threshold = 1.2). A gene was considered to have a recurrent G4 in a category if this share was at least T, and to be position-specific if it was at least T in one category and below T in all others. T was varied from 0.30 to 0.95 in steps of 0.05. The null distribution was obtained by 1000 permutations in which, within each species, the observed PQS patterns (the combination of the four categories) of PQS-carrying genes were reassigned at random among all genes with an *Arabidopsis* homolog in that species. This preserves the number of PQS-carrying genes per species, the frequency of each category and their co-occurrence, but removes gene identity. One-sided empirical *p*-values were calculated as (1 + number of permutations with a count ≥ observed)/(1000 + 1) [31]. Because species are not phylogenetically independent and the null model does not account for gene-level sequence properties (such as coding-sequence composition), this test shows non-random recurrence and does not distinguish selection from sequence conservation, and its results are descriptive.

### 4.8. Statistical Analysis

Statistical analyses were performed in Python 3.12.3 using SciPy 1.17.1 [32] and statsmodels 0.14.6 [33]. GO enrichment was tested with one-sided Fisher’s exact tests and Benjamini–Hochberg correction for multiple comparisons (FDR < 0.05; Section 4.5), and the gene-level permutation test is described in Section 4.7. The relationship between genomic GC content and the proportion of PQS-positive genes was assessed with Spearman’s rank correlation, and the difference in GC content and maximum G4Hunter score between genes with and without a homolog with a two-sided Wilcoxon signed-rank test on paired species medians.

### 4.9. Generative AI Declaration

**Figure 5:**
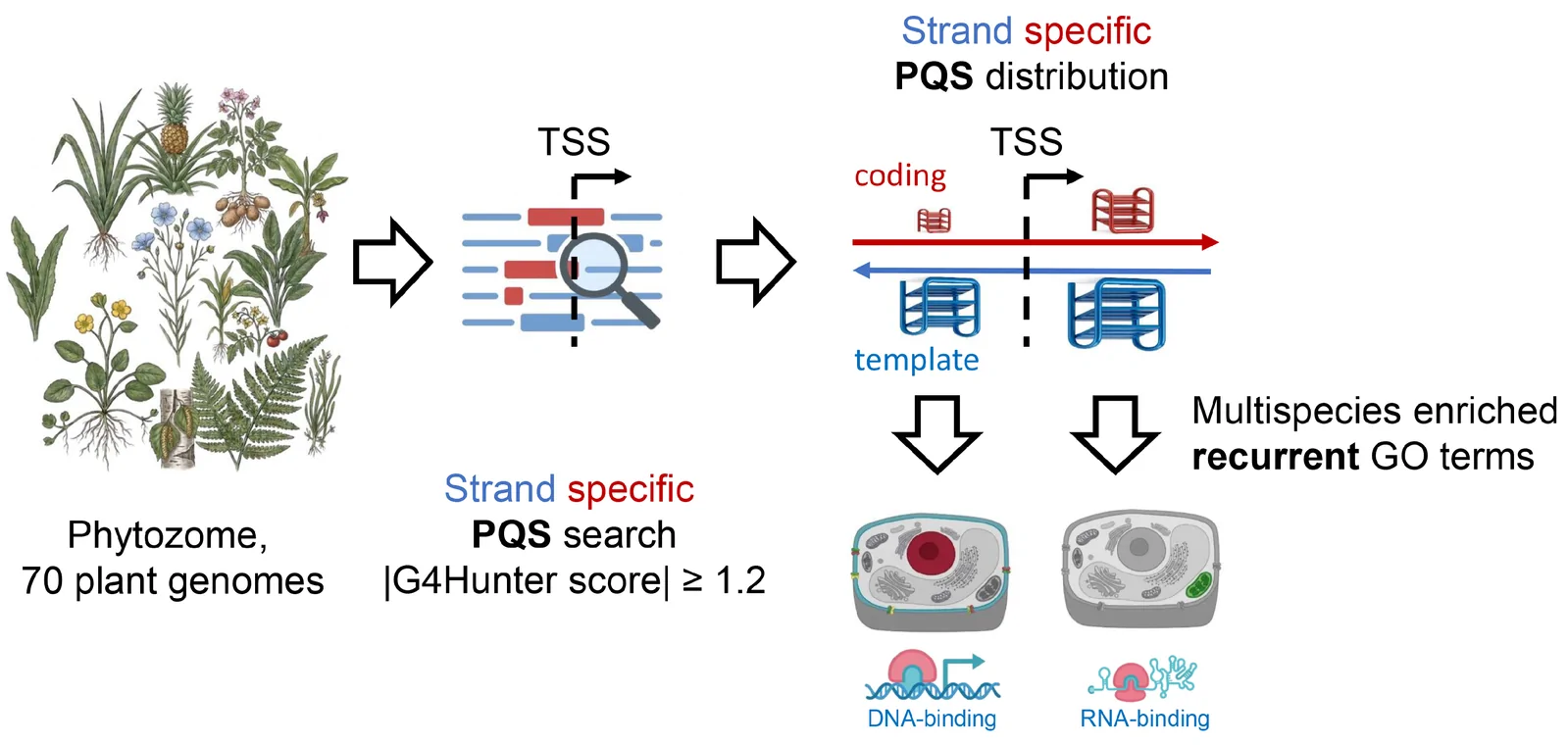
Schematic overview of the computational workflow for the strand-specific distribution of putative G4-forming sequences (PQS) and functional analysis across plant genomes.

Google Gemini (Google LLC, Mountain View, CA, USA) was used to generate illustrative icons for Figure 5. The authors are fully responsible for the content of the manuscript.

## 5. Conclusions

The main steps of the performed analysis and a summary of the obtained results are shown in Figure 5. By mapping putative G-quadruplex-forming sequences (PQS) across 70 plant species, we established a comparative framework relating G4 occurrence to genomic position, strand orientation, and gene function. The analysis revealed recurrent associations between G4 topology and functional gene classes: downstream template-strand motifs were associated with chloroplast, organellar, and RNA-binding functions, and upstream template-strand motifs with transcriptional regulation, whereas downstream coding-strand motifs showed a weaker signal. At the level of individual genes, a position-specific G4 recurred among homologs more often than expected at random, mostly in downstream regions, where this may partly reflect the conservation of the coding sequence; upstream genes with a recurrent G4 were fewer and included transcription factors.

These associations are based on computationally predicted PQS, modest enrichment effect sizes, and descriptive cross-species analyses and should be regarded as hypotheses. Experimental validation is required to establish the regulatory roles of G4 motifs in these gene groups.

The resulting map identifies gene classes and G4 configurations that can now be prioritized for experimental validation and for exploring the potential of G4-based cis-regulatory elements in plant biotechnology.

## Figures and Tables

**Table 1:** Gene Ontology (GO) terms recurrently enriched among genes with a G4 motif near the TSS in 70 plant species (G4Hunter threshold = 1.2).

GO ID	Name	Number of Species	Median FDR	Median Fold	UC	DC	UT	DT
GO:0009507	chloroplast	53	8.92 × 10^−9^	1.14	0	0	0	**53**
GO:0009570	chloroplast stroma	46	4.14 × 10^−5^	1.27	0	0	0	**46**
GO:0009941	chloroplast envelope	45	9.12 × 10^−5^	1.26	0	0	0	**45**
GO:0003729	mRNA binding	43	5.69 × 10^−4^	1.21	5	2	2	**42**
GO:0000976	transcription cis-regulatory region binding	57	1.62 × 10^−5^	1.40	24	33	**48**	10
GO:0003700	DNA-binding transcription factor activity	57	3.40 × 10^−6^	1.35	26	40	**50**	12
GO:0005515	protein binding	56	3.77 × 10^−4^	1.11	19	26	**39**	29
GO:0006355	regulation of DNA-templated transcription	52	5.93 × 10^−5^	1.33	22	30	**45**	8
GO:0005634	nucleus	42	1.31 × 10^−4^	1.08	15	24	**27**	10
GO:0071456	cellular response to hypoxia	36	4.29 × 10^−3^	1.60	1	4	**35**	4
GO:0043565	sequence-specific DNA binding	31	8.73 × 10^−3^	1.50	13	8	**22**	2
GO:0005886	plasma membrane	30	1.87 × 10^−3^	1.15	13	7	**27**	6
GO:0003677	DNA binding	27	1.09 × 10^−2^	1.38	4	6	**21**	2
GO:0009506	plasmodesma	26	1.07 × 10^−2^	1.27	4	1	**25**	0
GO:0004674	protein serine/threonine kinase activity	30	7.80 × 10^−3^	1.36	11	13	14	4

FDR—false discovery rate; UC—upstream coding; DC—downstream coding; UT—upstream template; DT—downstream template; Bold font indicates the predominant category of a term.

## Data Availability

All data analyzed in this study are publicly available from Phytozome v13, TAIR, and TimeTree; the derived data are provided in the Supplementary Materials. The custom scripts are available from the corresponding author upon reasonable request.

## Supplementary Materials

The following supporting information can be downloaded at: https://www.mdpi.com/article/10.3390/ijms27219823/s1.
