# Supplementary material for: Position and Strand Identity of G-Quadruplex Motifs Are Associated with Distinct Functional Signatures in Plants
Source: Int J Mol Sci. 2026 Nov 2;27(21):9823. doi: 10.3390/ijms27219823 (PMC13763612; doi:10.3390/ijms27219823)
Supplement: Supplementary file 1 [file ijms-27-09823-s001.zip › ijms-4625939-supplementary.pdf]

Supplementary Materials

Position and Strand Identity of G-Quadruplex Motifs Are Associated with Distinct Functional Signatures in Plants

Dmitry N. Kaluzhny, Daiana A. Krupskaya, Olesya D. Moskalenko, Elena N. Pushkova, Nataliya V. Melnikova, Alexey A. Dmitriev

Engelhardt Institute of Molecular Biology, Russian Academy of Sciences, 119991 Moscow, Russia

Table of content

Table S1. Genome assembly and annotation versions for the 70 plant species analyzed in this study.........2

Figure S1. G4 density profiles around transcription start sites (TSSs) across 70 plant species (G4Hunter threshold = 2.0)....................5

Figure S2. G4 density profiles around transcription start sites (TSSs) across 70 plant species (G4Hunter threshold = 1.8)....................6

Figure S3. G4 density profiles around transcription start sites (TSSs) across 70 plant species (G4Hunter threshold = 1.6)....................7

Figure S4. G4 density profiles around transcription start sites (TSSs) across 70 plant species (G4Hunter threshold = 1.4)....................8

Figure S5. G4 density profiles around transcription start sites (TSSs) across 70 plant species (G4Hunter threshold = 1.0)....................9

Figure S6. Filtering of BLASTp best hits used to assign *Arabidopsis thaliana* homologs to genes of 70 plant species. 10

Figure S7. Robustness of Gene Ontology (GO) enrichment to the G4Hunter threshold. 11

Figure S8. Gene Ontology (GO) enrichment of genes grouped by GC content and the status of putative G4-forming sequences (PQS) in the window around the transcription start site (TSS)....................12

Figure S9. Recurrence of position-specific G-quadruplexes at homologous genes across 70 plant species. 13

Table S2. Genes with a position-specific upstream G4 recurrent among their homologs....................14

Table S1. Genome assembly and annotation versions for the 70 plant species analyzed in this study.
All data were obtained from Phytozome v13 (https://phytozome-next.jgi.doe.gov, accessed on 15 September 2026).

Species	Phytozome short name	Assembly version	Annotation version	Phytozome genome ID	GC, %	Analyzed TSS
Acorus americanus	Aamericanus	v1.0	v1.1	586	38.44	26,508
Amaranthus hypochondriacus	Ahypochondriacus	v2.0	v2.1	459	32.65	23,847
Amborella trichopoda	Atrichopoda	v1.0	v1.0	291	35.45	26,846
Ananas comosus	Acomosus	v3	v3	321	37.66	27,024
Aquilegia coerulea	Acoerulea	v3	v3.1	322	35.18	30,023
Arabidopsis thaliana	Athaliana	TAIR10	Araport11	447	36.00	27,655
Arachis hypogaea	Ahypogaea	v1.0	v1.0	530	36.27	67,124
Asparagus officinalis	Aofficinalis	V1	V1.1	498	37.86	27,395
Beta vulgaris	Bvulgaris	EL10_1.0	EL10_1.0	548	35.85	24,255
Betula platyphylla	Bplatyphylla	v1.0	v1.1	679	35.80	33,967
Brassica rapa	BrapaFPsc	v1.0	v1.3	277	27.94	40,492
Camelina sativa	CsativaCAM116	v1.0	v1.1	802	36.68	74,852
Carya illinoinensis	Cillinoinensis	v1.0	v1.1	573	35.98	31,911
Castanea dentata	Cdentata	v1.0	v1.1	673	35.24	31,254
Ceratopteris richardii	Crichardii	v2.0	v2.1	676	38.40	36,857
Cercis canadensis	Ccanadensis	v3.0	V3.1	705	36.30	27,440
Chenopodium quinoa	Cquinoa	v1.0	v1.0	392	35.19	44,776
Chlamydomonas reinhardtii	Creinhardtii	v5.0	v5.5	281	61.74	17,741
Cicer arietinum	Carietinum	v1.0	v1.0	492	28.15	28,269
Citrus sinensis	Csinensis	v1.0	v1.1	154	27.39	25,379
Coffea arabica	Carabica	v0.5	v0.5	453	36.90	70,830
Corylus avellana	Cavellanacv_JeffersonHAP1	v4.0	v4.0	858	36.36	32,431
Cucumis sativus	Csativus	v1.0	v1.0	122	30.58	21,503
Daucus carota	Dcarota	v2.0	v2.0	388	31.92	32,113
Dioscorea alata	Dalata	v1.0	v1.1	504	32.51	21,728
Diphasiastrum complanatum	Dcomplanatum	v3.0	v3.1	723	38.42	31,430

Eucalyptus grandis	Egrandis	v2.0	v2.0	297	36.39	36,349
Fragaria vesca	Fvesca	v2.0.a1	v2.0.a2	501	35.69	33,538
Glycine max	Gmax	v2.0	Wm82.a2.v1	275	33.94	56,044
Gossypium barbadense	Gbarbadense	v1.0	v1.1	526	34.09	74,561
Helianthus annuus	Hannuus	r1.0	r1.2	494	37.22	52,243
Hydrangea quercifolia	Hquercifolia	v1.0	v1.1	589	36.30	31,139
Hydrocotyle leucocephala	HleucocephalaHAP2	v2.0	v2.1	769	34.70	32,135
Kalanchoe fedtschenkoi	Kfedtschenkoi	v1.0	v1.1	382	34.47	30,964
Lactuca sativa	Lsativa	V8	v5	467	34.80	38,910
Lens culinaris	Lculinaris	v1	v1	718	36.90	39,141
Linum usitatissimum	Lusitatissimum	v1.0	v1.0	200	37.56	43,471
Liriodendron tulipifera	LtulipiferaYP108A	v1.0	v1.1	766	41.36	29,655
Lupinus albus	Lalbus	r1	v1	567	33.54	38,258
Malus domestica	Mdomestica	GDDH13v1.1	v1.1	491	33.49	45,116
Manihot esculenta	Mesculenta	v6.1	v6.1	305	30.58	33,033
Marchantia polymorpha	Mpolymorpha	v3.0	v3.1	320	39.14	19,287
Medicago truncatula	Mtruncatula	Mt4.0	Mt4.0v1	285	31.25	50,894
Mimulus guttatus	Mguttatus	v2.0	v2.0	256	32.84	28,140
Musa acuminata	Macuminata	v1	v1	304	32.09	36,528
Nymphaea colorata	Ncolorata	v1.0	v1.2	566	38.60	24,059
Olea europaea	Oeuropaea	v1.0	v1.0	451	31.97	50,684
Oryza sativa	Osativa	v7.0	v7.0	323	43.55	42,189
Physcomitrium patens	Ppatens	v3	v3.3	318	33.29	32,926
Populus deltoides	PdeltoidesWV94	v2.0	v2.1	445	32.32	44,853
Porphyra umbilicalis	Pumbilicalis	v1.5	v1.5	456	65.68	13,125
Portulaca amilis	Pamilis	v1.0	v1.0	692	36.73	52,037
Quercus rubra	Qrubra	v2.0	v2.1	687	34.54	33,333
Ricinus communis	Rcommunis	v0.1	v0.1	119	32.52	31,221
Salix purpurea	Spurpurea	v1.0	v1.0	289	24.89	37,865
Saponaria officinalis	Sofficinalis	v1.0	v1.1	780	39.18	37,604

Selaginella moellendorffii	Smoellendorffii	v1.0	v1.0	91	44.40	22,285
Solanum lycopersicum	Slycopersicum	SL4.0	ITAG4.0	691	34.34	34,075
Solanum tuberosum	Stuberosum	v4.03	v4.03	448	30.45	39,028
Sorghum bicolor	Sbicolor	v3.0.1	v3.1.1	454	41.81	34,129
Sphagnum magellanicum	Smagellanicum	v1.0	v1.1	521	36.25	25,227
Theobroma cacao	Tcacao	v1.1	v1.1	233	32.52	29,452
Thuja plicata	Tplicata	v3.0	v3.1	572	31.32	39,659
Typha latifolia	Tlatifoliavar_var. 2019.6	v1.0	v1.1	853	37.72	22,107
Ulmus americana	Uamericanavar_NA87034HAP1	v1.0	v1.1	881	34.81	25,621
Vaccinium darrowii	Vdarrowii	v1.2	v1.2	700	38.64	34,809
Vigna unguiculata	Vunguiculata	v1.0	v1.1	469	32.82	29,773
Vitis vinifera	Vvinifera	Genoscope.12X	v2.1	457	33.41	31,845
Zea mays	Zmays	APGv4	RefGen_V4	493	46.19	39,498
Zostera marina	Zmarina	v2.2	v2.2	324	36.03	20,450

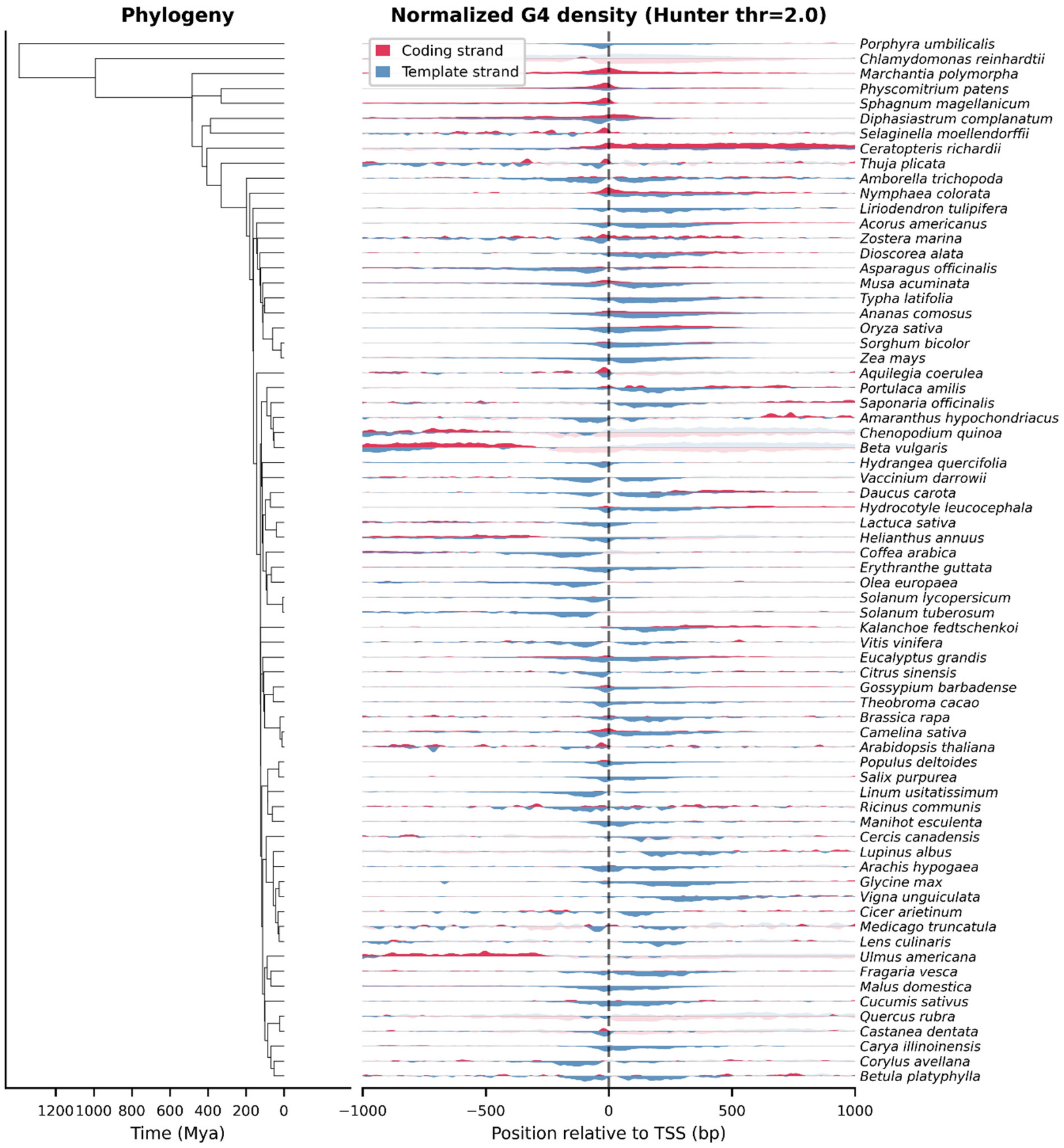

Figure S1. G4 density profiles around transcription start sites (TSSs) across 70 plant species (G4Hunter threshold = 2.0).
Color coding and track representation follow Figure 1 of the main manuscript.

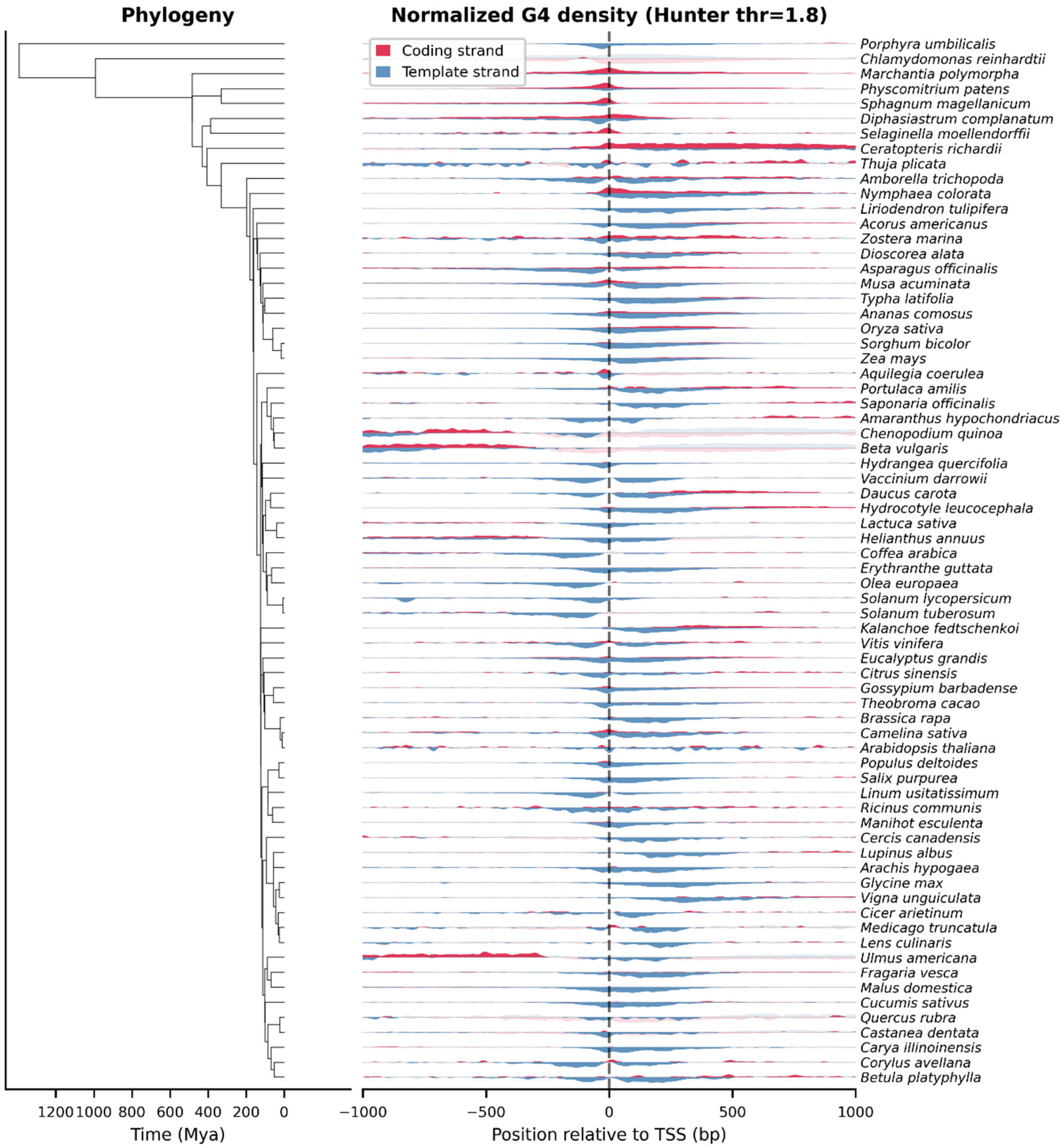

Figure S2. G4 density profiles around transcription start sites (TSSs) across 70 plant species (G4Hunter threshold = 1.8).

Color coding and track representation follow Figure 1 of the main manuscript.

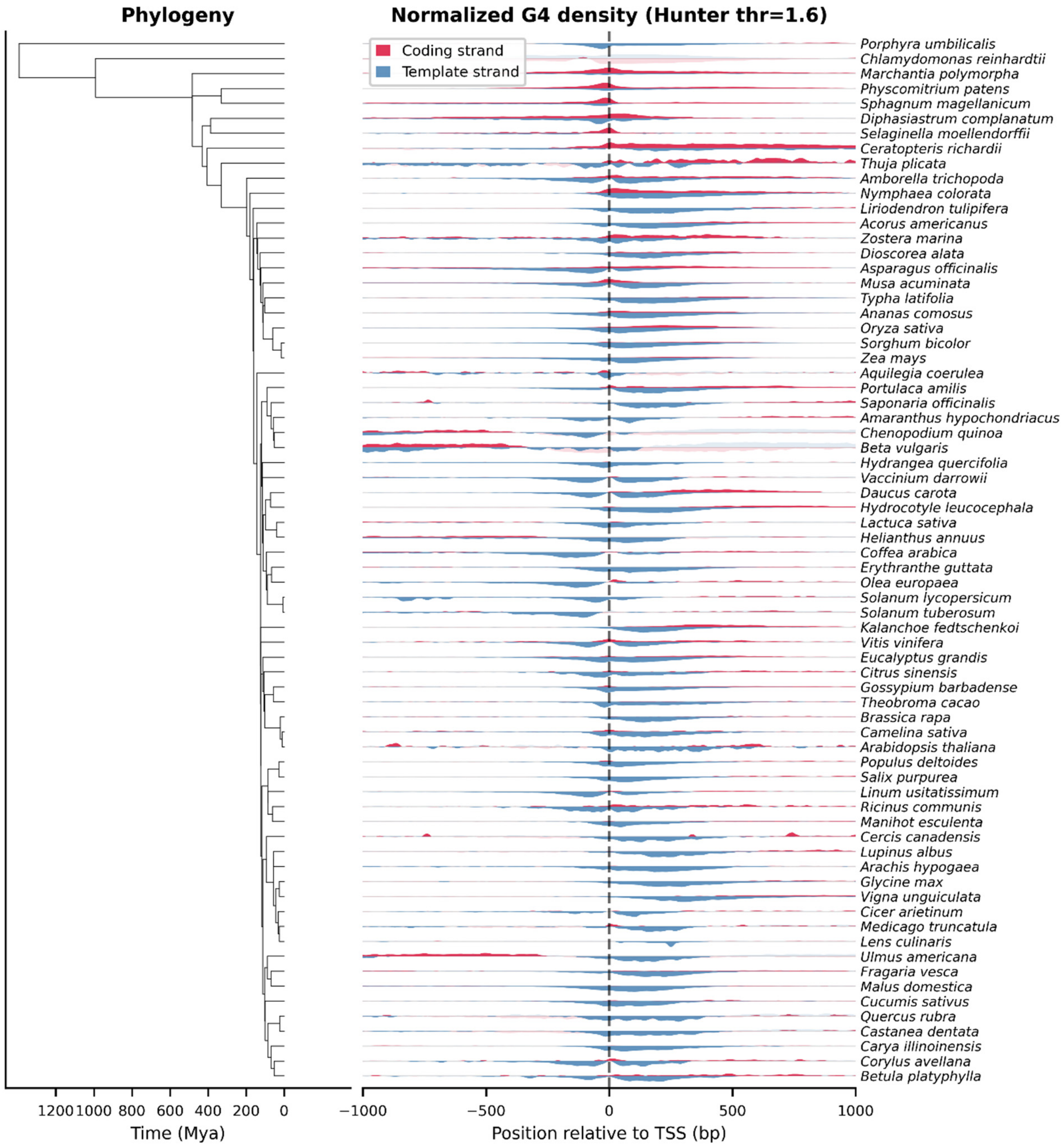

Figure S3. G4 density profiles around transcription start sites (TSSs) across 70 plant species (G4Hunter threshold = 1.6).
Color coding and track representation follow Figure 1 of the main manuscript.

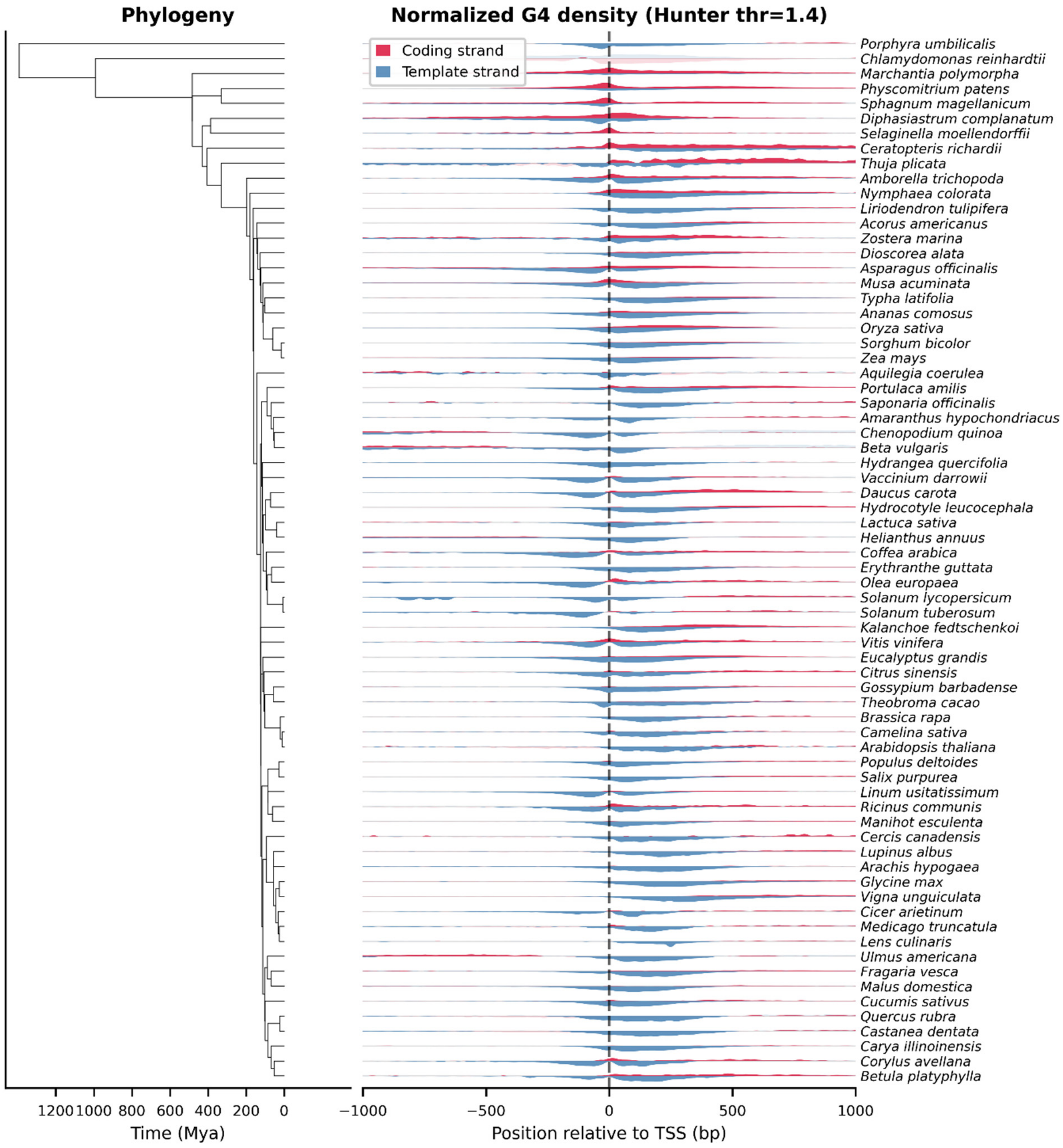

Figure S4. G4 density profiles around transcription start sites (TSSs) across 70 plant species (G4Hunter threshold = 1.4).
Color coding and track representation follow Figure 1 of the main manuscript.

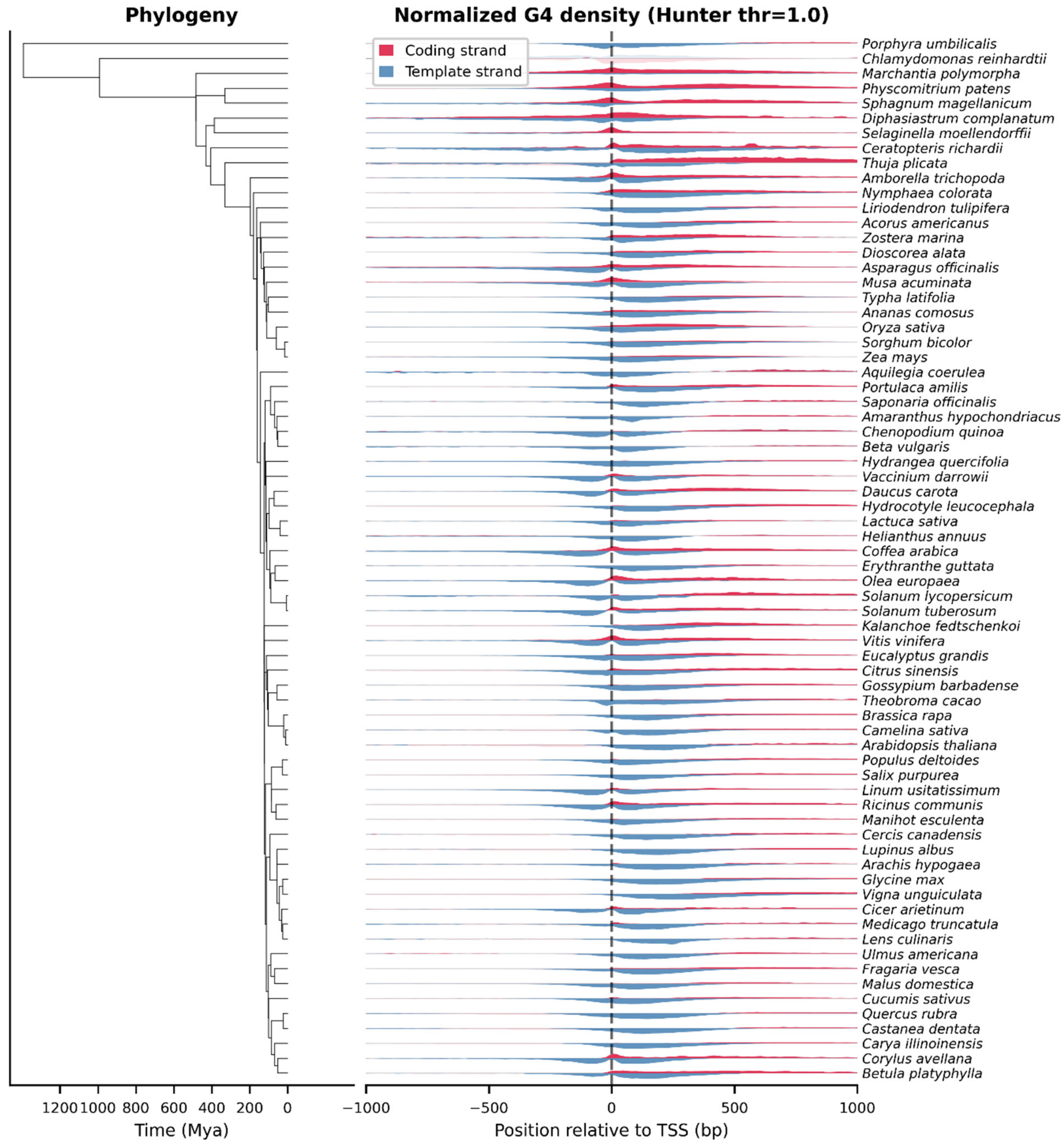

Figure S5. G4 density profiles around transcription start sites (TSSs) across 70 plant species (G4Hunter threshold = 1.0).
Color coding and track representation follow Figure 1 of the main manuscript.

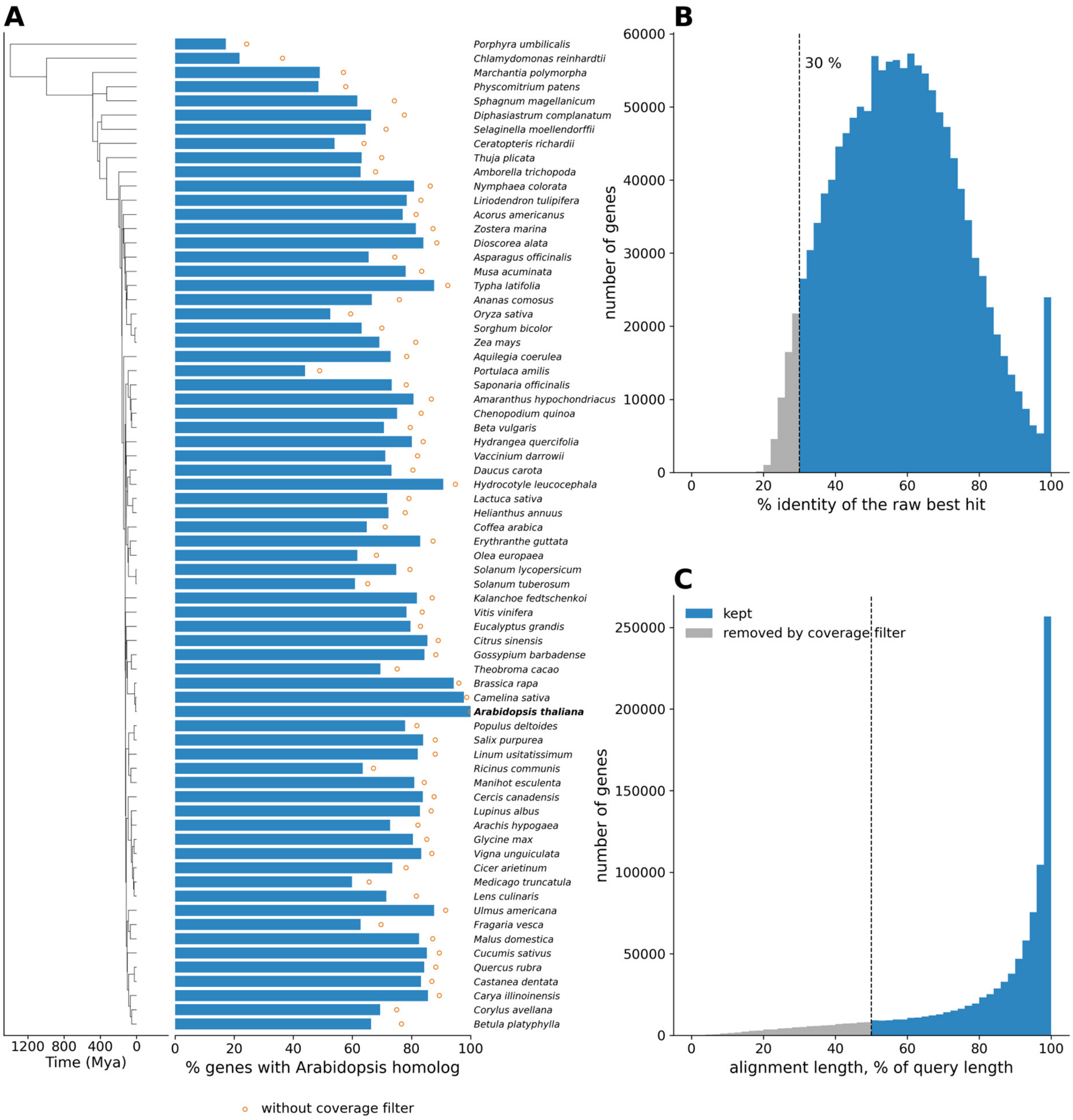

Figure S6. Filtering of BLASTp best hits used to assign *Arabidopsis thaliana* homologs to genes of 70 plant species. Each gene (longest transcript) was searched with BLASTp against the *A. thaliana* proteome (TAIR10; E-value ≤ 1e-5, best hit only, one local alignment per query). Identity and coverage criteria were applied after the search: a hit was accepted as a homolog if it had ≥ 30% sequence identity and the alignment covered ≥ 50% of the query length. (**A**) The percentage of genes of each species with an accepted *A. thaliana* homolog (blue bars); open orange circles show the percentage obtained without the coverage criterion. The *A. thaliana* bar (bold) reflects the self-hit. (**B**) Distribution of the percentage identity of the raw best hits (E ≤ 1e-5), pooled across all species; hits below the 30% identity cut-off (dashed line) are shown in gray. The peak at 100% corresponds to *A. thaliana* matched to itself. (**C**) Distribution of the alignment length as a percentage of the query length for hits passing the identity criterion; hits removed by the 50% coverage criterion (dashed line) are shown in gray.

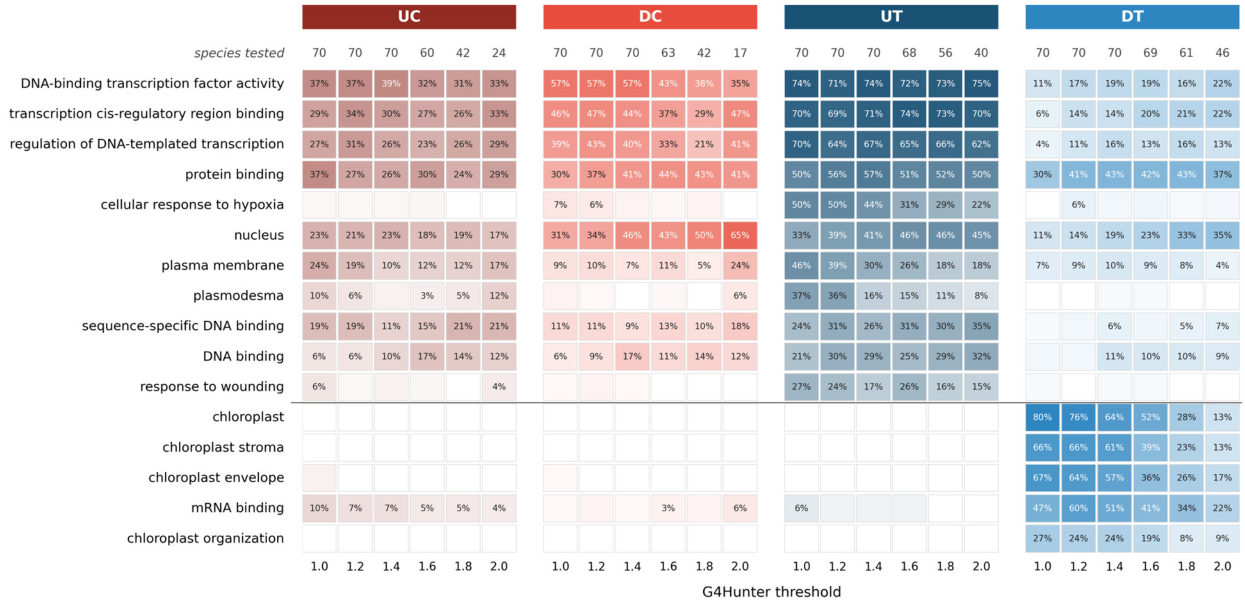

Figure S7. Robustness of Gene Ontology (GO) enrichment to the G4Hunter threshold.

Rows: GO terms enriched in ≥ 15 species in at least one position/strand category at a threshold of 1.2 (maximum 20 terms). Columns: Position/strand categories (UC, DC, UT, DT), each at G4Hunter thresholds of 1.0–2.0. Cell color and value: percentage of tested species in which the term is significantly enriched (FDR < 0.05, species-specific background); values below 3% are not labeled. The top row shows the number of species tested (≥ 300 GO-annotated genes in the category at that threshold), which decreases at stricter thresholds.

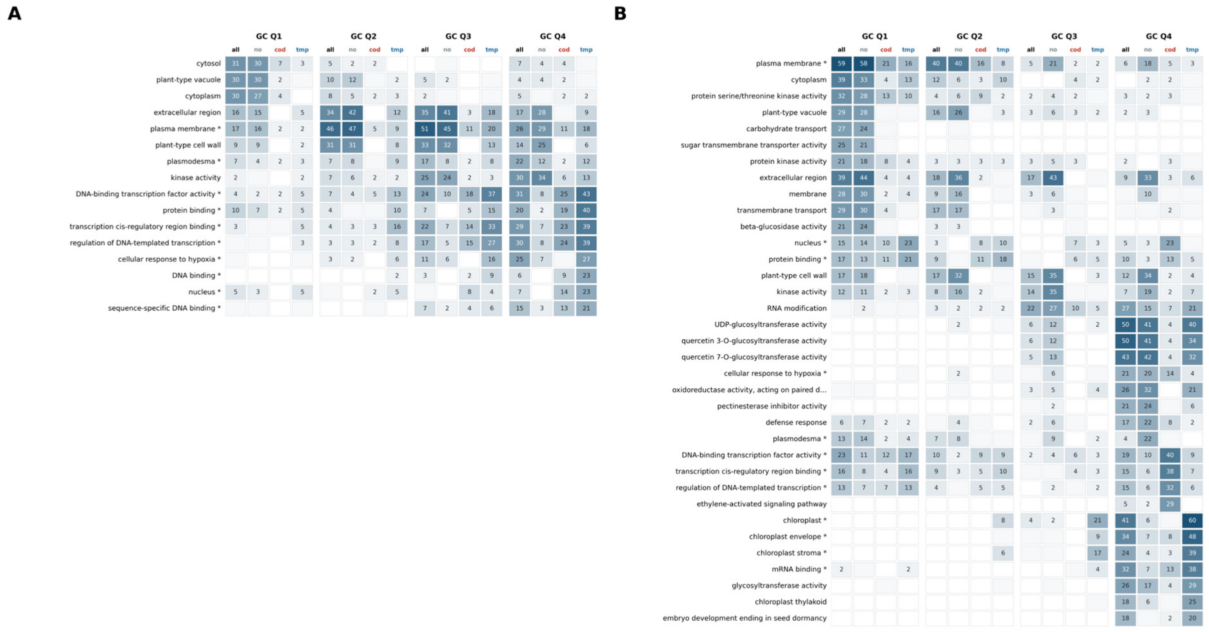

Figure S8. Gene Ontology (GO) enrichment of genes grouped by GC content and the status of putative G4-forming sequences (PQS) in the window around the transcription start site (TSS).

(**A**) Upstream window (−500…−1 bp); (**B**) Downstream window (0…+499 bp). In each species, genes were divided into four equal GC-content quartiles of the respective window (Q1, lowest; Q4, highest). Within each quartile, GO enrichment was tested for all genes of the quartile (all), genes without PQS on either strand (no), genes with PQS on the coding strand (cod), and genes with PQS on the template strand (tmp); PQS were identified with G4Hunter at a threshold of 1.2. The cod and tmp groups are not mutually exclusive. Each group was tested against the background of all genes of species that have an *Arabidopsis thaliana* homolog, so genes of the other groups and quartiles were part of the background. Cell values show the number of species (out of 70) with significant enrichment (one-sided Fisher's exact test, Benjamini-Hochberg FDR < 0.05); a group was tested in a species only if it contained at least 100 GO-annotated genes. Asterisks mark terms from Table 1 of the main manuscript.

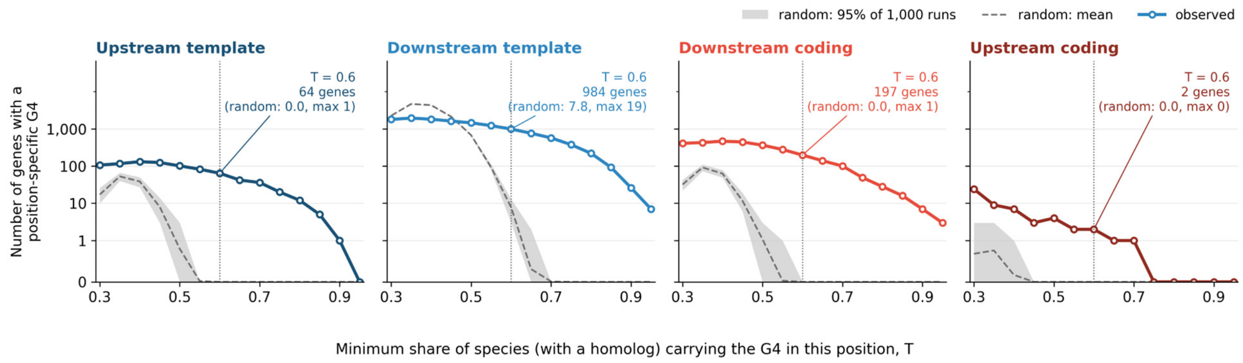

Figure S9. Recurrence of position-specific G-quadruplexes at homologous genes across 70 plant species.
For each position/strand category (UT, DT, DC, UC), the number of *Arabidopsis* genes with a homolog in at least 61 of the 70 species whose homologs carry a G4 in that category in at least a share T of the species, and in no other category at that level (colored line), plotted against T. The gray area and dashed line show the number obtained when G4-carrying genes were randomly reassigned among the genes of each species (95% of 1,000 permutations and the mean). Dotted line: T = 0.6, with the number of genes and the random mean and maximum. G4Hunter threshold = 1.2.

Table S2. Genes with a position-specific upstream G4 recurrent among their homologs.
Arabidopsis genes (≥ 61 species with a homolog) in which the homologs carry a G4 in the upstream position/strand category (UT, upstream template; UC, upstream coding) in ≥ 60% of the species and in no other category at that level (T = 0.6; G4Hunter threshold = 1.2). Species with a homolog: Number of species with a detected homolog. UC, DC, UT, DT: Share of these species in which the homolog carries a G4 in the category.

***Arabidopsis* gene**	**Symbol**	**Species with a homolog**	**UC**	**DC**	**UT**	**DT**	**TAIR GO terms (recurrent)**
AT1G15690	AtAVP1	70	0.41	0.43	0.67	0.57	chloroplast; plasma membrane; chloroplast envelope; mRNA binding
AT1G32060	PRK	70	0.19	0.26	0.66	0.51	chloroplast stroma; protein binding; chloroplast envelope; chloroplast; nucleus
AT2G21790	ATRNR1	70	0.13	0.26	0.61	0.59	
AT5G44790	AtHMP51	70	0.24	0.51	0.64	0.46	
AT3G11320		69	0.26	0.35	0.62	0.59	plasma membrane
AT4G13940	ATSAHH1	69	0.17	0.23	0.77	0.59	plasma membrane; protein binding; mRNA binding; plasmodesma
AT4G34050	CCoAOMT1	69	0.22	0.17	0.72	0.55	nucleus; plasma membrane
AT1G70600	uL15x	68	0.19	0.57	0.75	0.54	plasmodesma; mRNA binding
AT2G03500	EFM	68	0.38	0.37	0.66	0.51	protein binding; regulation of DNA-templated transcription; DNA-binding transcription factor activity; transcription cis-regulatory region binding; nucleus
AT2G26710	BAS1	68	0.43	0.43	0.62	0.56	
AT3G02130	CLI1	68	0.29	0.54	0.63	0.53	plasma membrane; protein binding
AT3G56370	IRK	68	0.21	0.31	0.60	0.47	protein binding; plasma membrane
AT4G21450		68	0.32	0.37	0.63	0.53	plasma membrane; nucleus; protein binding
AT5G19730	PME53	68	0.28	0.38	0.63	0.50	chloroplast
AT5G45340	CYP707A3	68	0.28	0.16	0.65	0.49	chloroplast; cellular response to hypoxia
AT5G53460	GLT1	68	0.31	0.44	0.62	0.41	chloroplast stroma; chloroplast
AT1G08280	GALT29A	67	0.22	0.28	0.67	0.52	protein binding
AT3G18290	BTS	67	0.28	0.58	0.61	0.49	protein binding; cellular response to hypoxia; nucleus
AT4G21200	ATGA2OX8	67	0.24	0.45	0.63	0.46	
AT5G02500	AT-HSC70-1	67	0.43	0.43	0.75	0.49	plasmodesma; chloroplast; nucleus; protein binding; plasma membrane; mRNA binding
AT5G11970		67	0.16	0.48	0.63	0.54	

AT5G62000	ARF1-BP	67	0.45	0.49	0.67	0.46	protein binding; regulation of DNA-templated transcription; nucleus; transcription cis-regulatory region binding; DNA-binding transcription factor activity; sequence-specific DNA binding
AT1G01720	ANAC002	66	0.24	0.38	0.76	0.47	regulation of DNA-templated transcription; DNA-binding transcription factor activity; nucleus; transcription cis-regulatory region binding; response to wounding; cellular response to hypoxia
AT1G78700	BEH4	66	0.33	0.52	0.71	0.56	regulation of DNA-templated transcription; nucleus; DNA-binding transcription factor activity
AT2G32070	CAF1k	66	0.27	0.27	0.64	0.55	nucleus; protein binding
AT3G12600	atnudt16	66	0.24	0.36	0.64	0.52	nucleus
AT3G22600	LTPG5	66	0.21	0.45	0.64	0.58	
AT3G45980	H2B	66	0.33	0.45	0.79	0.47	DNA binding; chloroplast stroma; nucleus
AT4G13420	ATHAK5	66	0.33	0.56	0.64	0.48	nucleus
AT5G67360	ARA12	66	0.36	0.50	0.61	0.56	
AT1G26480	GF14 IOTA	65	0.31	0.26	0.63	0.54	nucleus
AT1G44110	CYCA1;1	65	0.20	0.34	0.68	0.38	protein binding; nucleus
AT2G41900	AtC3H30	65	0.29	0.42	0.60	0.51	nucleus; DNA-binding transcription factor activity; regulation of DNA-templated transcription; protein binding
AT1G32450	AtNPF7.3	64	0.31	0.23	0.64	0.56	plasma membrane; nucleus
AT1G75390	AtbZIP44	64	0.16	0.31	0.78	0.42	regulation of DNA-templated transcription; transcription cis-regulatory region binding; protein binding; nucleus; sequence-specific DNA binding; DNA-binding transcription factor activity
AT1G75450	ATCKX5	64	0.28	0.48	0.70	0.56	
AT2G46150		64	0.27	0.55	0.64	0.53	plasma membrane
AT3G57040	ARR9	64	0.50	0.41	0.72	0.59	nucleus; protein binding; regulation of DNA-templated transcription; sequence-specific DNA binding; DNA-binding transcription factor activity
AT4G25810	XTH23	64	0.34	0.52	0.77	0.52	
AT5G01710		64	0.22	0.31	0.64	0.59	
AT1G65910	anac028	63	0.41	0.17	0.62	0.49	regulation of DNA-templated transcription; DNA-binding transcription factor activity; transcription cis-regulatory region binding; nucleus
AT3G18280	AtTED4	63	0.22	0.25	0.70	0.51	
AT3G22970		63	0.24	0.29	0.63	0.54	nucleus; chloroplast

AT4G15800	RALF33	63	0.22	0.57	0.65	0.54	response to wounding; plasmodesma
AT5G20510	AL5	63	0.24	0.38	0.62	0.56	nucleus; DNA binding; transcription cis-regulatory region binding
AT5G53950	ANAC098	63	0.22	0.27	0.68	0.41	transcription cis-regulatory region binding; nucleus; regulation of DNA-templated transcription; DNA-binding transcription factor activity; protein binding
AT1G28110	SCPL45	62	0.19	0.29	0.61	0.56	chloroplast; plasmodesma
AT1G70710	ATGH9B1	62	0.23	0.52	0.65	0.60	chloroplast
AT2G01300		62	0.21	0.56	0.69	0.50	chloroplast
AT3G04730	IAA16	62	0.18	0.52	0.65	0.55	protein binding; transcription cis-regulatory region binding; regulation of DNA-templated transcription; nucleus; DNA-binding transcription factor activity
AT3G07350		62	0.11	0.29	0.69	0.39	cellular response to hypoxia
AT4G10265	WIP3	62	0.31	0.50	0.68	0.47	nucleus; cellular response to hypoxia
AT4G35750		62	0.27	0.21	0.61	0.50	
AT5G04800	eS17w	62	0.18	0.34	0.61	0.44	mRNA binding; plasma membrane
AT1G75250	ATRL6	61	0.31	0.30	0.80	0.59	regulation of DNA-templated transcription; nucleus; DNA-binding transcription factor activity
AT1G80840	ATWRKY40	61	0.23	0.20	0.61	0.41	protein binding; nucleus; transcription cis-regulatory region binding; DNA-binding transcription factor activity; response to wounding; regulation of DNA-templated transcription
AT2G42800	AtRLP29	61	0.10	0.20	0.66	0.57	plasma membrane
AT3G23160		61	0.34	0.49	0.62	0.57	
AT3G55240	BPG4	61	0.41	0.15	0.72	0.38	nucleus; chloroplast; protein binding
AT3G62420	ATBZIP53	61	0.20	0.26	0.62	0.39	protein binding; DNA-binding transcription factor activity; nucleus; sequence-specific DNA binding; transcription cis-regulatory region binding
AT4G32480		61	0.30	0.48	0.64	0.26	cellular response to hypoxia; nucleus
AT5G47550	CYS5	61	0.23	0.57	0.69	0.57	
AT5G57685	ATGDU3	61	0.16	0.39	0.67	0.48	protein binding; nucleus
AT5G65160	TPR14	61	0.21	0.46	0.64	0.52	
AT4G13710	ATPLL25	68	0.66	0.41	0.51	0.43	
AT2G29420	ATGSTU7	63	0.60	0.52	0.57	0.54	
